# Supplementary material for: Varying relationships between experienced income segregation and travel behaviour across neighbourhood social and urban contexts
Source: Nat Commun. 2025 Dec 18;16:11236. doi: 10.1038/s41467-025-66585-z (PMC12714732; doi:10.1038/s41467-025-66585-z)
Supplement: Supplementary file 1 — Supplementary Information [file 41467_2025_66585_MOESM1_ESM.pdf]

Varying relationships between experienced income segregation and travel behaviour across neighbourhood social and urban contexts

Yuxuan Zhou[1], Yi Lu[1,2]*

[1] Department of Architecture and Civil Engineering, City University of Hong Kong, Hong Kong Special Administrative Region, China

[2] City University of Hong Kong Shenzhen Research Institute, Shenzhen, China

*Corresponding author, Yi Lu (yilu24@cityu.edu.hk)

List of Supplementary Items

Supplementary Table 1 1

Supplementary Table 2 2

Supplementary Table 3 3

Supplementary Table 4 4

Supplementary Table 5 5

Supplementary Table 6 6

Supplementary Table 7 7

Supplementary Table 8 8

Supplementary Table 9 9

Supplementary Table 10 10

Supplementary Table 11 11

Supplementary Fig. 1 12

Supplementary Fig. 2 13

Supplementary Fig. 3 14

Supplementary Fig. 4 15

Supplementary Fig. 5 16

Supplementary Fig. 6 17

Supplementary Fig. 7 18

Supplementary Fig. 8 19

Supplementary Fig. 9 20

Supplementary Fig. 10 21

Supplementary Fig. 11 22

Supplementary Fig. 12 23

Supplementary Fig. 13 24

Supplementary Fig. 14 25

Supplementary Fig. 15 26

Supplementary Fig. 16 27

Supplementary Fig. 17 28

Supplementary Fig. 18 29

Supplementary Fig. 19 30

Supplementary Fig. 20 31
Supplementary Fig. 21 32
Supplementary Fig. 22 33
Supplementary Fig. 23 34
Supplementary Fig. 24 35
Supplementary Fig. 25 36
Supplementary Fig. 26 37
Supplementary Fig. 27 38
Supplementary Fig. 28 39
Supplementary Fig. 29 40
Supplementary Fig. 30 41

Supplementary Table 1. North American Industry Classification System (NAICS) codes of selected point of interest (POI) categories

Category	NAICS code
Culture	712110, 712120
Entertainment	711110, 711120, 711130, 711190, 711211, 711212, 711219, 711310, 711320, 711410, 711510, 713110, 713120, 713210, 713290
Grocery	445110, 445120
Healthcare	62111, 622112, 621210, 621310, 621320, 621330, 621340, 621391, 621399, 621410, 621420, 621491, 621492, 621493, 621498
Hospital	622110, 622210, 622310
Hotel	721110, 721120, 721191, 721199
Life service	485310, 485320, 492110, 811111, 811112, 811113, 811118, 811121, 811122, 811191, 811192, 811198, 811211, 811212, 811213, 811219, 811310, 811411, 811412, 811420,811430, 811490, 812310, 811411, 811412, 811420, 811430, 811490, 812310, 812320, 812910, 812921, 812922
Market	445110, 445220, 445230
Park	712190
Personal care	812111, 812112, 812113, 812191, 812199
Religious	813110
Restaurant	722511, 722513, 722514, 722515
School	611110, 611210, 611310
Social assistance	624110, 624120, 624190, 624210, 624221, 624229, 624230, 624310, 624410
Sport	713920, 713940, 713950
Shopping	442110, 442210, 442291, 442299, 443141, 443142, 444120, 444130, 444210, 444220, 445291, 445292, 445299, 445310, 446110, 446120, 446130, 446191, 446199, 448110, 448120, 448130, 448140, 448150, 448190, 448210, 448310, 448320, 451110, 451120, 451130, 451140, 451211, 451212, 452210, 452311, 452319, 453110, 453220, 453310, 453910, 453991, 453998

Supplementary Table 2. The number of origin-destination (OD) pairs for each point of interest (POI) category

Category	Number of POIs	Number of OD pairs
Culture	13,783	5,951,556
Entertainment	8,622	16,070,690
Grocery	117,663	69,222,348
Healthcare	440,164	56,860,243
Hospital	9,268	15,754,406
Hotel	66,931	35,740,224
Life service	213,571	14,495,375
Market	25,691	6,372,342
Park	96,914	46,624,091
Personal care	136,040	8,145,761
Religious	210,902	26,336,458
Restaurant	705,007	433,049,500
School	149,312	70,734,688
Social assistance	123,182	22,444,767
Sport	122,885	60,932,939
Shopping	787,907	337,714,887

Supplementary Table 3. Description of the primary Rural–Urban Commuting Area (RUCA) codes

Code	Description
1	Metropolitan area core: primary flow within an urbanized area (UA)
2	Metropolitan area high commuting: primary flow 30% or more to a UA
3	Metropolitan area low commuting: primary flow 10% to 30% to a UA
4	Micropolitan area core: primary flow within an urban cluster of 10,000 to 49,999 (large UC)
5	Micropolitan high commuting: primary flow 30% or more to a large UC
6	Micropolitan low commuting: primary flow 10% to 30% to a large UC
7	Small town core: primary flow within an urban cluster of 2,500 to 9,999 (small UC)
8	Small town high commuting: primary flow 30% or more to a small UC
9	Small town low commuting: primary flow 10% to 30% to a small UC
10	Rural areas: primary flow to a tract outside a UA or UC

Supplementary Table 4. Multivariate linear regression model results for the association between average travel distance and experienced segregation interacting with income quartiles at the neighbourhood level. Statistical significance was assessed using two-sided tests.

	Metropolitan areas (p-value)	Micropolitan areas (p-value)	Small towns (p-value)	Rural areas (p-value)
Travel distance	-0.219 (0.000)	-0.171 (0.000)	-0.198 (0.000)	-0.324 (0.000)
Travel distance × Income quartile 2 (ref. Income quartile 1)	0.208 (0.000)	0.010 (0.643)	0.019 (0.504)	0.142 (0.000)
Travel distance × Income quartile 3 (ref. Income quartile 1)	0.299 (0.000)	0.107 (0.000)	0.128 (0.000)	0.220 (0.000)
Travel distance × Income quartile 4 (ref. Income quartile 1)	0.359 (0.000)	0.401 (0.000)	0.336 (0.000)	0.451 (0.000)
Income quartile 2 (ref. Income quartile 1)	-0.194 (0.000)	-0.217 (0.000)	-0.258 (0.000)	-0.312 (0.000)
Income quartile 3 (ref. Income quartile 1)	0.466 (0.000)	-0.258 (0.000)	-0.368 (0.000)	-0.321 (0.000)
Income quartile 4 (ref. Income quartile 1)	1.485 (0.000)	-0.779 (0.000)	-0.882 (0.000)	-0.579 (0.000)
Population density	-0.065 (0.000)	-0.144 (0.000)	-0.107 (0.000)	-0.094 (0.000)
Median age	0.011 (0.000)	0.025 (0.001)	0.016 (0.094)	-0.047 (0.000)
Proportion of females	-0.016 (0.000)	-0.004 (0.512)	-0.022 (0.015)	0.018 (0.075)
Proportion of white people	0.002 (0.401)	-0.116 (0.000)	-0.050 (0.000)	0.050 (0.000)
Proportion of people with high school diploma	0.080 (0.000)	0.086 (0.000)	0.075 (0.000)	0.098 (0.000)
Proportion of tenures with access to at least one vehicle	0.055 (0.000)	0.038 (0.000)	0.032 (0.002)	-0.035 (0.002)
Observations	167,780	20,580	10,847	8,683
Adjusted R^2	0.453	0.139	0.143	0.111

Supplementary Table 5. Multivariate linear regression model results for the association between travel diversity and experienced segregation interacting with income quartiles at the neighbourhood level. Statistical significance was assessed using two-sided tests.

	Metropolitan areas (p-value)	Micropolitan areas (p-value)	Small towns (p-value)	Rural areas (p-value)
Travel diversity	-0.017 (0.000)	-0.225 (0.000)	-0.318 (0.000)	-0.365 (0.000)
Travel diversity × Income quartile 2 (ref. Income quartile 1)	-0.126 (0.000)	-0.040 (0.019)	0.032 (0.178)	0.212 (0.000)
Travel diversity × Income quartile 3 (ref. Income quartile 1)	0.105 (0.000)	0.346 (0.000)	0.416 (0.000)	0.604 (0.000)
Travel diversity × Income quartile 4 (ref. Income quartile 1)	0.133 (0.000)	0.649 (0.000)	0.817 (0.000)	0.795 (0.000)
Income quartile 2 (ref. Income quartile 1)	-0.310 (0.000)	-0.231 (0.000)	-0.211 (0.00)	-0.187 (0.000)
Income quartile 3 (ref. Income quartile 1)	0.381 (0.000)	-0.243 (0.000)	-0.302 (0.000)	-0.237 (0.000)
Income quartile 4 (ref. Income quartile 1)	1.460 (0.000)	-0.739 (0.000)	-0.889 (0.000)	-0.551 (0.000)
Population density	-0.065 (0.000)	-0.115 (0.000)	-0.075 (0.000)	-0062 (0.000)
Median age	0.009 (0.000)	0.027 (0.000)	0.005 (0.572)	-0.058 (0.000)
Proportion of females	-0.015 (0.000)	-0.005 (0.412)	-0.016 (0.069)	0.029 (0.004)
Proportion of white people	-0.004 (0.068)	-0.138 (0.000)	-0.063 (0.000)	0.035 (0.001)
Proportion of people with high school diploma	0.067 (0.000)	0.089 (0.000)	0.082 (0.000)	0.087 (0.000)
Proportion of tenures with access to at least one vehicle	0.029 (0.000)	0.006 (0.390)	-0.001 (0.887)	-0.054 (0.000)
Observations	167,780	20,580	10,847	8,683
Adjusted R^2	0.448	0.182	0.223	0.163

Supplementary Table 6. Multivariate linear regression model results for the association between travel distance and experienced segregation interacting with residential segregation among low-income neighbourhoods (neighbourhoods with low residential segregation: n=41,945 in metropolitan areas, n=5,145 in micropolitan areas, n=2,712 in small towns, n=2,174 in rural areas). Statistical significance was assessed using two-sided tests.

	Metropolitan areas (p-value)	Micropolitan areas (p-value)	Small towns (p-value)	Rural areas (p-value)
Travel distance	-0.072 (0.000)	-0.068 (0.000)	-0.180 (0.000)	-0.265 (0.000)
Travel distance × Low residential segregation (ref. High residential segregation)	0.022 (0.000)	-0.101 (0.000)	0.015 (0.479)	0.103 (0.000)
Low residential segregation (ref. High residential segregation)	-0.853 (0.000)	-0.847 (0.000)	-1.039 (0.000)	-1.037 (0.000)
Median household income	-0.120 (0.000)	-0.092 (0.000)	-0.112 (0.000)	-0.170 (0.000)
Population density	-0.102 (0.000)	-0.246 (0.000)	-0.184 (0.000)	-0.104 (0.000)
Median age	0.035 (0.000)	0.049 (0.000)	0.023 (0.039)	-0.032 (0.014)
Proportion of females	-0.022 (0.000)	-0.003 (0.679)	-0.028 (0.009)	0.019 (0.121)
Proportion of white people	0.049 (0.000)	-0.186 (0.000)	-0.092 (0.000)	0.050 (0.000)
Proportion of people with high school diploma	0.135 (0.000)	0.029 (0.000)	0.023 (0.031)	0.054 (0.000)
Proportion of tenures with access to at least one vehicle	0.096 (0.000)	0.092 (0.000)	0.068 (0.000)	0.024 (0.067)
Observations	83,890	10,290	5,424	4,348
Adjusted R^2	0.280	0.345	0.405	0.406

Supplementary Table 7. Multivariate linear regression model results for the association between travel diversity and experienced segregation interacting with residential segregation among low-income neighbourhoods (neighbourhoods with low residential segregation: n=41,945 in metropolitan areas, n=5,145 in micropolitan areas, n=2,712 in small towns, n=2,174 in rural areas). Statistical significance was assessed using two-sided tests.

	Metropolitan areas (p-value)	Micropolitan areas (p-value)	Small towns (p-value)	Rural areas (p-value)
Travel diversity	-0.071 (0.000)	-0.170 (0.000)	-0.139 (0.000)	-0.163 (0.000)
Travel diversity × Low residential segregation (ref. High residential segregation)	-0.071 (0.000)	-0.077 (0.000)	-0.126 (0.000)	0.003 (0.892)
Low residential segregation (ref. High residential segregation)	-0.858 (0.000)	-0.816 (0.000)	-0.979 (0.000)	-1.014 (0.000)
Median household income	-0.124 (0.000)	-0.083 (0.000)	-0.100 (0.000)	-0.145 (0.000)
Population density	-0.125 (0.000)	-0.179 (0.000)	-0.125 (0.000)	-0.070 (0.000)
Median age	0.021 (0.000)	0.043 (0.000)	0.003 (0.797)	-0.084 (0.000)
Proportion of females	-0.015 (0.000)	0.009 (0.271)	-0.013 (0.203)	0.034 (0.004)
Proportion of white people	0.019 (0.000)	-0.201 (0.000)	-0.086 (0.000)	0.069 (0.000)
Proportion of people with high school diploma	0.147 (0.000)	0.046 (0.000)	0.038 (0.000)	0.075 (0.000)
Proportion of tenures with access to at least one vehicle	0.080 (0.000)	0.048 (0.000)	0.028 (0.018)	0.009 (0.494)
Observations	83,890	10,290	5,424	4,348
Adjusted R^2	0.288	0.367	0.418	0.388

Supplementary Table 8. Multivariate linear regression model results for the association between travel distance and experienced segregation interacting with racial composition among low-income neighbourhoods (majority-POC neighbourhoods : n=24,749 in metropolitan areas, n=1,371 in micropolitan areas, n=785 in small towns, n=434 in rural areas; majority-White neighbourhoods: 59,141 in metropolitan areas, n=8,919 in micropolitan areas, n=4,639 in small towns, n=3,914 in rural areas). Statistical significance was assessed using two-sided tests.

	Metropolitan areas (p-value)	Micropolitan areas (p-value)	Small towns (p-value)	Rural areas (p-value)
Travel distance	-0.177 (0.000)	-0.157 (0.000)	-0.250 (0.000)	-0.225 (0.000)
Travel distance ×majority White (ref. majority POC)	0.120 (0.000)	0.007 (0.809)	0.072 (0.041)	-0.024 (0.560)
Low residential segregation (ref. majority POC)	0.012 (0.136)	-0.666 (0.000)	-0.419 (0.000)	-0.034 (0.515)
Median household income	-0.241 (0.000)	-0.191 (0.000)	-0.193 (0.000)	-0.251 (0.000)
Population density	-0.100 (0.000)	-0.239 (0.000)	-0.213 (0.000)	-0.134 (0.000)
Median age	0.029 (0.000)	0.042 (0.000)	0.036 (0.006)	-0.012 (0.430)
Proportion of females	-0.045 (0.000)	-0.018 (0.050)	-0.042 (0.001)	0.002 (0.888)
Proportion of people with high school diploma	0.170 (0.000)	0.031 (0.001)	0.018 (0.171)	0.076 (0.000)
Proportion of tenures with access to at least one vehicle	0.125 (0.000)	0.109 (0.000)	0.087 (0.000)	0.035 (0.004)
Observations	83,890	10,290	5,424	4,348
Adjusted R^2	0.122	0.175	0.145	0.145

Supplementary Table 9. Multivariate linear regression model results for the association between travel diversity and experienced segregation interacting with racial composition among low-income neighbourhoods (majority-POC neighbourhoods : n=24,749 in metropolitan areas, n=1,371 in micropolitan areas, n=785 in small towns, n=434 in rural areas; majority-White neighbourhoods: 59,141 in metropolitan areas, n=8,919 in micropolitan areas, n=4,639 in small towns, n=3,914 in rural areas). Statistical significance was assessed using two-sided tests.

	Metropolitan areas (p-value)	Micropolitan areas (p-value)	Small towns (p-value)	Rural areas (p-value)
Travel diversity	0.043 (0.000)	0.117 (0.000)	0.026 (0.440)	-0.216 (0.000)
Travel diversity ×majority White (ref. majority POC)	-0.242 (0.000)	-0.426 (0.000)	-0.375 (0.000)	-0.033 (0.496)
Low residential segregation (ref. majority POC)	-0.044 (0.000)	-0.685 (0.000)	-0.450 (0.000)	-0.022 (0.674)
Median household income	-0.237 (0.000)	-0.168 (0.000)	-0.147 (0.000)	-0.198 (0.000)
Population density	-0.095 (0.000)	-0.153 (0.000)	-0.118 (0.000)	-0.088 (0.000)
Median age	0.009 (0.010)	0.032 (0.001)	0.022 (0.086)	-0.069 (0.000)
Proportion of females	-0.039 (0.000)	-0.006 (0.490)	-0.024 (0.045)	0.021 (0.135)
Proportion of people with high school diploma	0.182 (0.000)	0.048 (0.000)	0.029 (0.016)	0.103 (0.000)
Proportion of tenures with access to at least one vehicle	0.097 (0.000)	0.057 (0.000)	0.033 (0.016)	0.013 (0.416)
Observations	83,890	10,290	5,424	4,348
Adjusted R^2	0.139	0.233	0.209	0.146

Supplementary Table 10. Multivariate linear regression model results for the association between travel distance and experienced segregation interacting with access to public transits among low-income neighbourhoods (neighbourhoods with 15-minute transits: n=47,469 in metropolitan areas, n=850 in micropolitan areas, n=203 in small towns, n=56 in rural areas; neighbourhoods without 15-minute transits: n=36,421 in metropolitan areas, n=9,440 in micropolitan areas, n=5,221 in small towns, n=4,292 in rural areas). Statistical significance was assessed using two-sided tests.

	Metropolitan areas (p-value)	Micropolitan areas (p-value)	Small towns (p-value)	Rural areas (p-value)
Travel distance	-0.029 (0.000)	-0.131 (0.000)	-0.181 (0.000)	-0.245 (0.000)
Travel distance × 15-minute public transits (ref. No 15-minute transits)	-0.129 (0.000)	-0.041 (0.109)	-0.067 (0.304)	0.275 (0.003)
15-minute public transits (ref. No 15-minute transits)	-0.558 (0.000)	-0.692 (0.000)	-0.583 (0.000)	-0.608 (0.000)
Median household income	-0.234 (0.000)	-0.168 (0.000)	-0.184 (0.000)	-0.258 (0.000)
Population density	-0.063 (0.000)	-0.202 (0.000)	-0.190 (0.000)	-0.134 (0.000)
Median age	0.004 (0.256)	0.065 (0.000)	0.053 (0.000)	-0.026 (0.095)
Proportion of females	-0.044 (0.000)	-0.019 (0.037)	-0.045 (0.000)	0.002 (0.897)
Proportion of white people	-0.036 (0.000)	-0.230 (0.000)	-0.168 (0.000)	0.027 (0.098)
Proportion of people with high school diploma	0.123 (0.000)	0.026 (0.004)	0.013 (0.295)	0.070 (0.000)
Proportion of tenures with access to at least one vehicle	0.071 (0.000)	0.094 (0.000)	0.082 (0.000)	0.027 (0.088)
Observations	83,890	10,290	5,424	4,348
Adjusted R^2	0.186	0.211	0.162	0.151

Supplementary Table 11. Multivariate linear regression model results for the association between travel diversity and experienced segregation interacting with access to public transits among low-income neighbourhoods (neighbourhoods with 15-minute transits: n=47,469 in metropolitan areas, n=850 in micropolitan areas, n=203 in small towns, n=56 in rural areas; neighbourhoods without 15-minute transits: n=36,421 in metropolitan areas, n=9,440 in micropolitan areas, n=5,221 in small towns, n=4,292 in rural areas). Statistical significance was assessed using two-sided tests.

	Metropolitan areas (p-value)	Micropolitan areas (p-value)	Small towns (p-value)	Rural areas (p-value)
Travel diversity	-0.154 (0.000)	-0.243 (0.000)	-0.296 (0.000)	-0.240 (0.000)
Travel diversity × 15-minute public transits (ref. No 15-minute transits)	0.157 (0.000)	0.308 (0.000)	0.357 (0.000)	0.170 (0.220)
15-minute public transits (ref. No 15-minute transits)	-0.529 (0.000)	-0.804 (0.000)	-0.772 (0.000)	-0.607 (0.000)
Median household income	-0.242 (0.000)	-0.156 (0.000)	-0.155 (0.000)	-0.210 (0.000)
Population density	-0.051 (0.000)	-0.129 (0.000)	-0.107 (0.000)	-0.089 (0.000)
Median age	-0.013 (0.000)	0.057 (0.000)	0.028 (0.028)	-0.085 (0.000)
Proportion of females	-0.039 (0.000)	-0.005 (0.588)	-0.028 (0.023)	0.021 (0.141)
Proportion of white people	-0.064 (0.000)	-0.254 (0.000)	-0.165 (0.000)	0.039 (0.014)
Proportion of people with high school diploma	0.151 (0.000)	0.049 (0.000)	0.029 (0.018)	0.098 (0.000)
Proportion of tenures with access to at least one vehicle	0.060 (0.000)	0.046 (0.000)	0.027 (0.048)	0.004 (0.783)
Observations	83,890	10,290	5,424	4,348
Adjusted R^2	0.182	0.241	0.208	0.151

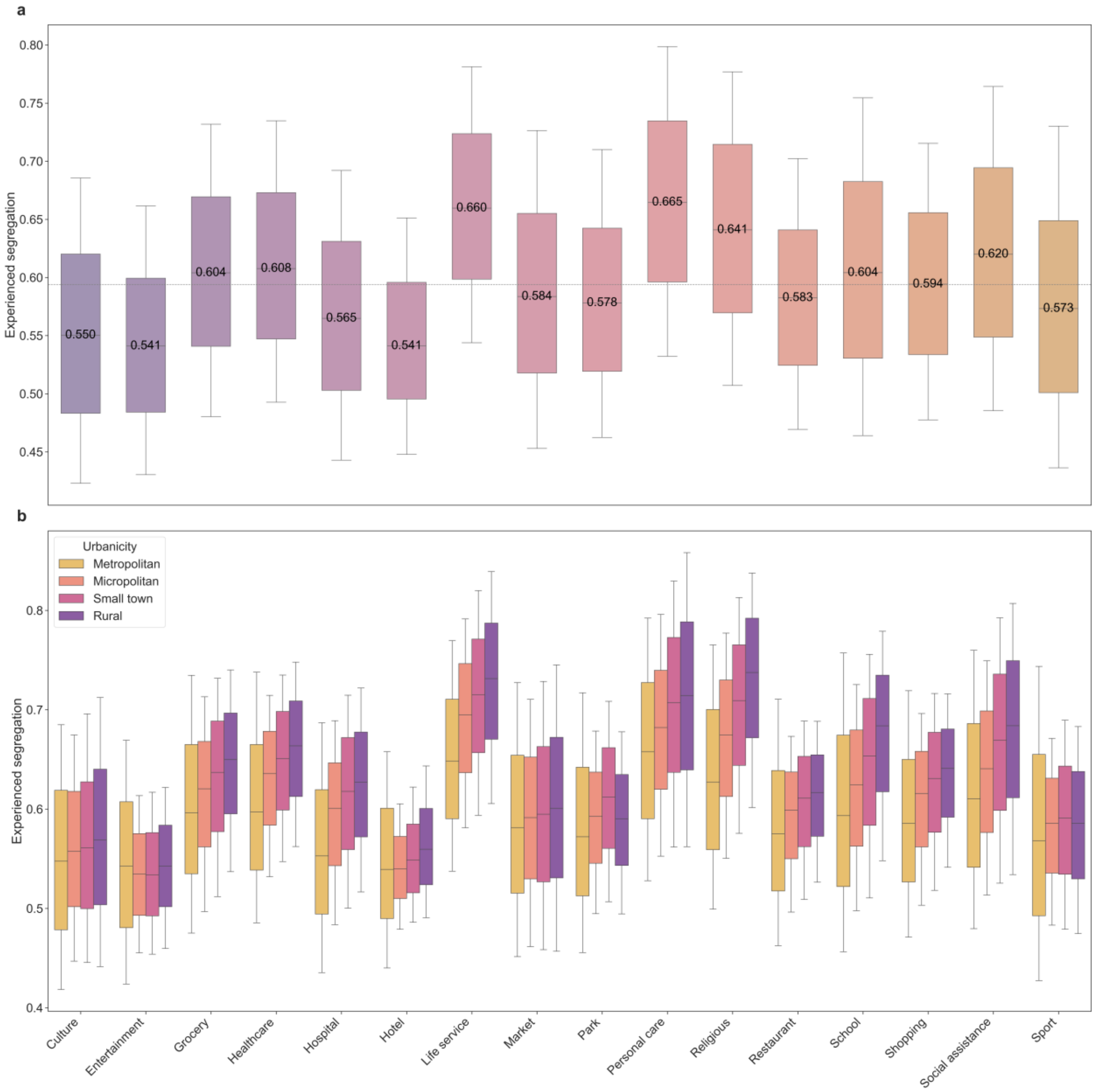

Supplementary Fig. 1 Experienced segregation by types of activity sites. a. Experienced segregation by types of activity sites (n=207,890 neighbourhoods). The dashed line represents the median values of the overall experienced segregation. **b.** Experienced segregation by types of activity sites across urbanicity levels (n=167,780, 20,580, 10,847, 8,683 neighbourhoods in metropolitan areas, micropolitan areas, small towns, and rural areas, respectively). The box plots in **a**, **b** present the 10th, 25th, 50th, 75th, and 90th percentiles.

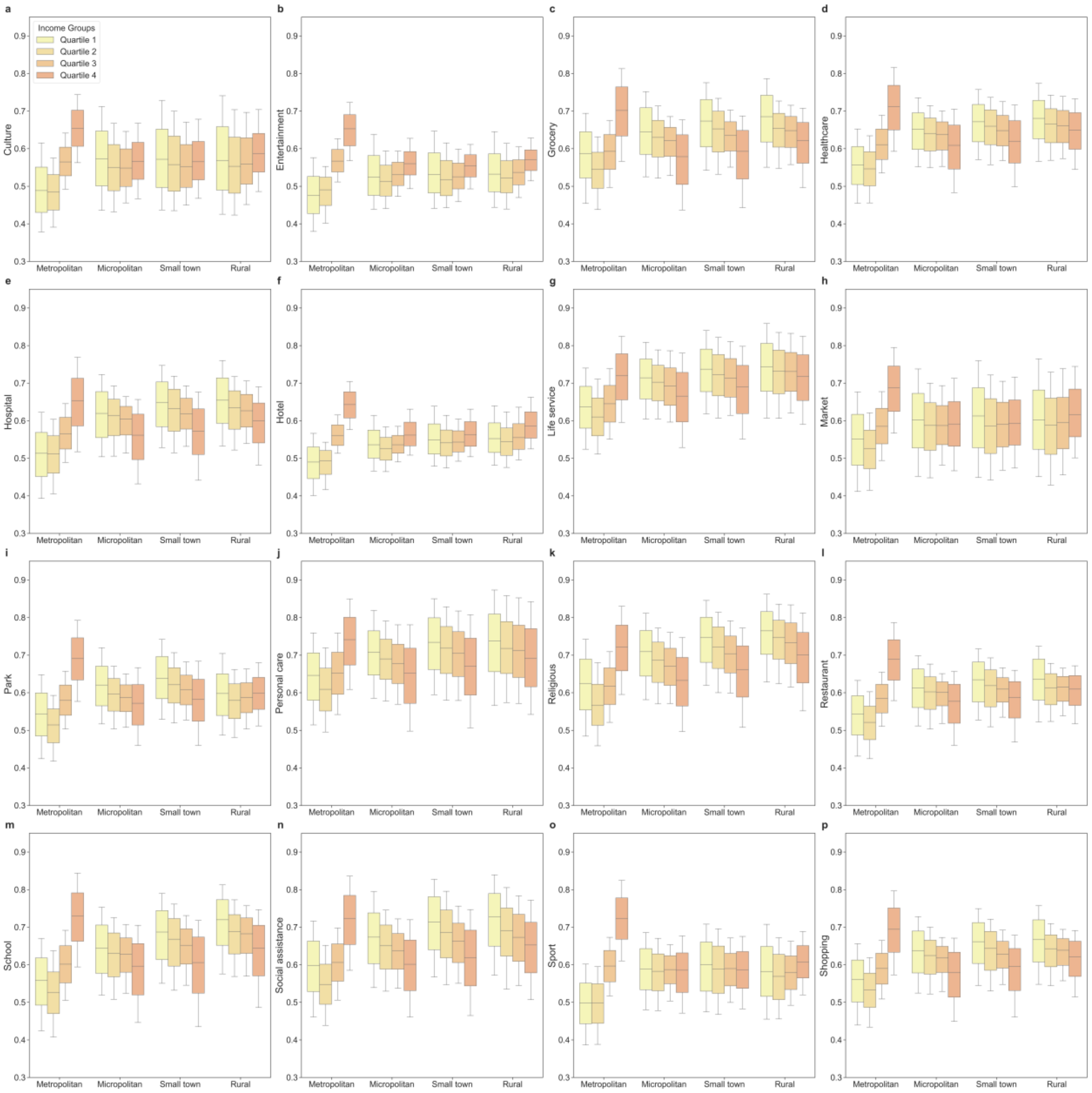

Supplementary Fig. 2 Experienced segregation by types of activity sites across income groups and urbanicity levels. In **a-p**, n=167,780, 20,580, 10,847, 8,683 neighbourhoods in metropolitan areas, micropolitan areas, small towns, and rural areas, respectively. The box plots in **a-p** present the 10th, 25th, 50th, 75th, and 90th percentiles.

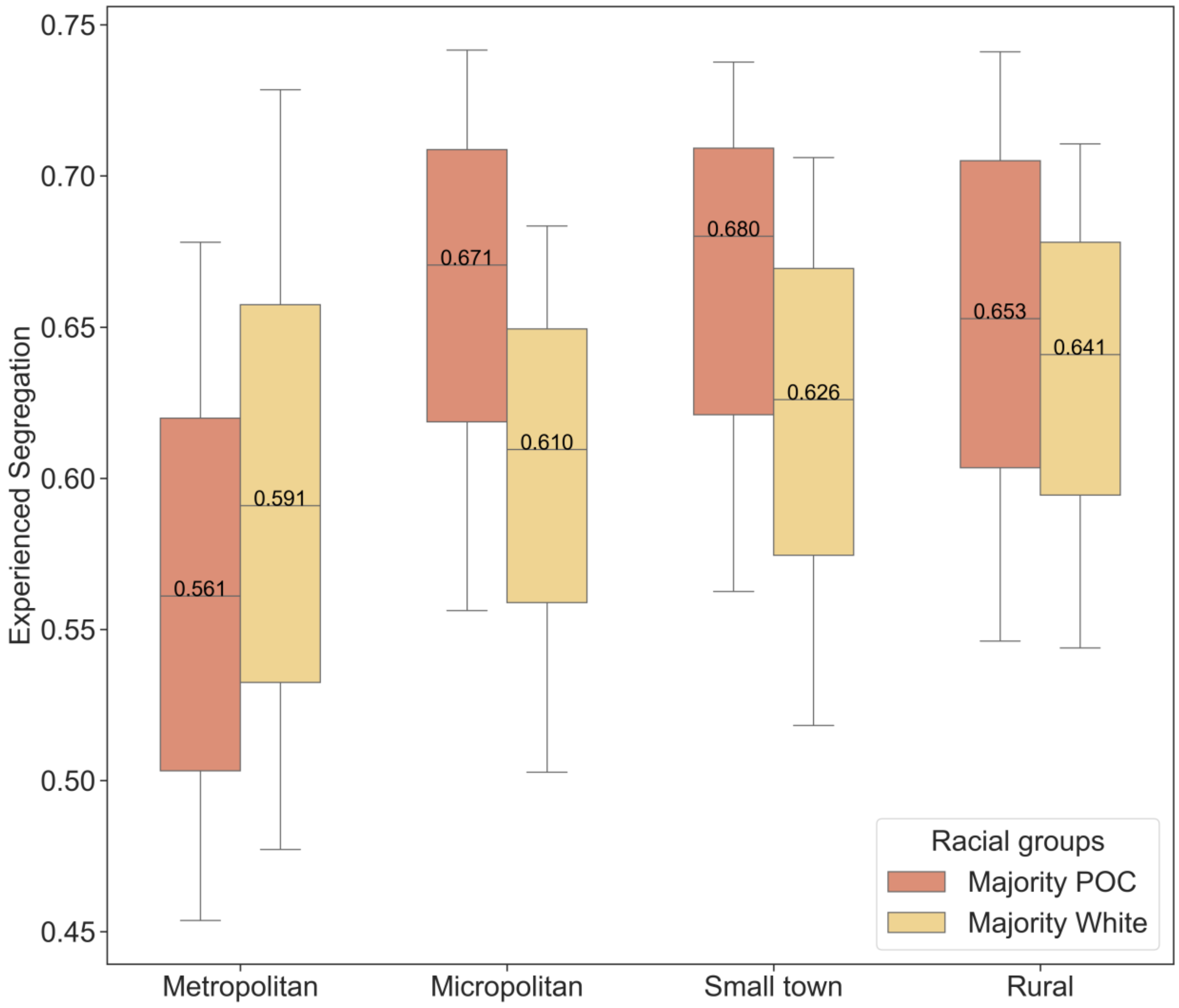

Supplementary Fig. 3 Distribution of overall experienced segregation between majority-POC (people of colour) and majority-White neighbourhoods across urbanicity levels. In metropolitan areas, n=34,468 majority-POC neighbourhoods, and 133,312 majority-White neighbourhoods. In micropolitan areas, n=1,523 majority-POC neighbourhoods, and 19,057 majority-White neighbourhoods. In small towns, n=911 majority-POC neighbourhoods, and 9,936 majority-White neighbourhoods. In rural areas, n=489 majority-POC neighbourhoods, and 8,194 majority-White neighbourhoods. The box plots present the 10th, 25th, 50th, 75th, and 90th percentiles.

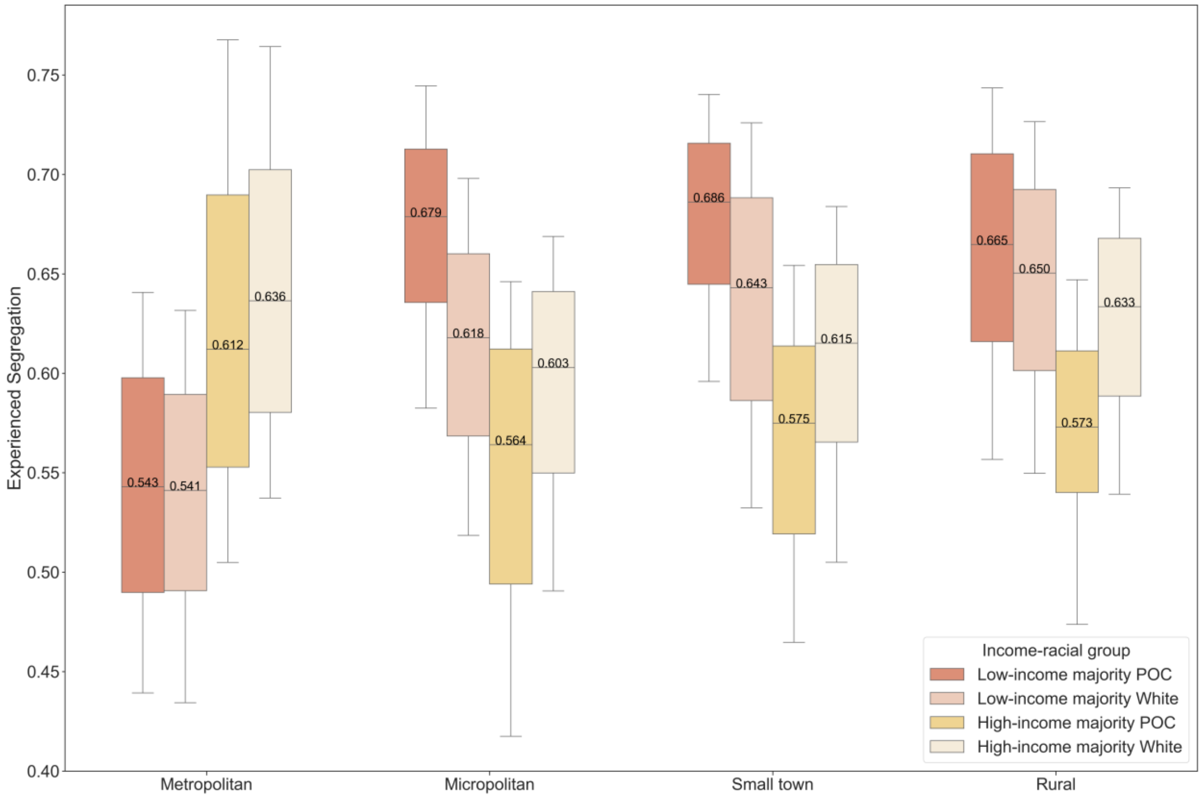

Supplementary Fig. 4 Distribution of overall experienced segregation across income-racial groups. Neighbourhoods in the 1st and 2nd income quartiles were designated as low-income, and those in the 3rd and 4th quartiles as high-income. These were then cross-classified by race composition into: (1) low-income, majority-POC (people of colour); (2) low-income, majority-White; (3) high-income, majority-POC; and (4) high-income, majority-White neighbourhoods. In metropolitan areas, n=24,749, 59,141, 9,719, and 74,171 neighbourhoods were classified as Low-income majority-POC, low-income majority-White, high-income majority-POC, and high-income majority-White. In micropolitan areas, n=1,371, 8,919, 152, and 10,138 neighbourhoods were classified as the same groups. In small towns, n=785, 4,639, 126, and 5,297 neighbourhoods were classified as the same groups. In rural areas, n=434, 3,914, 55, and 4,280 neighbourhoods were classified as the same groups. The box plots present the 10th, 25th, 50th, 75th, and 90th percentiles.

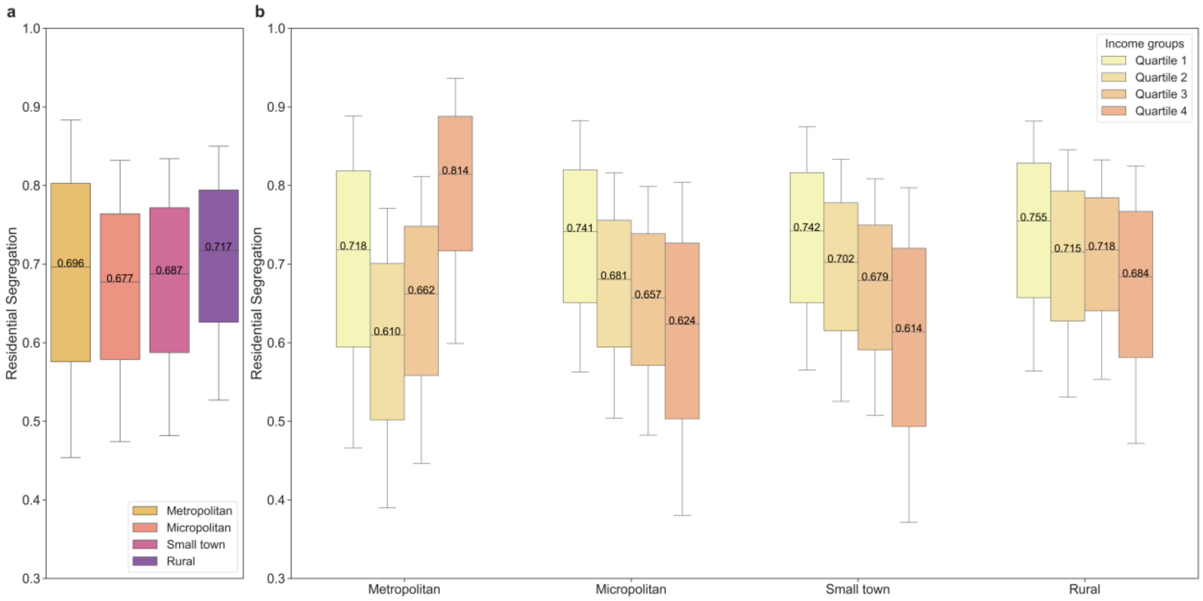

Supplementary Fig. 5 Distribution of residential segregation across urbanicity levels and income groups. a. Distribution of residential segregation across different urbanicity levels **b.** Distribution of residential segregation across various income quartiles in distinct urbanicity levels, with income quartiles defined separately for each urbanicity level. In **a**, **b**, n=167,780, 20,580, 10,847, 8,683 neighbourhoods in metropolitan areas, micropolitan areas, small towns, and rural areas, respectively. The box plots in **a**, **b** present the 10th, 25th, 50th, 75th, and 90th percentiles.

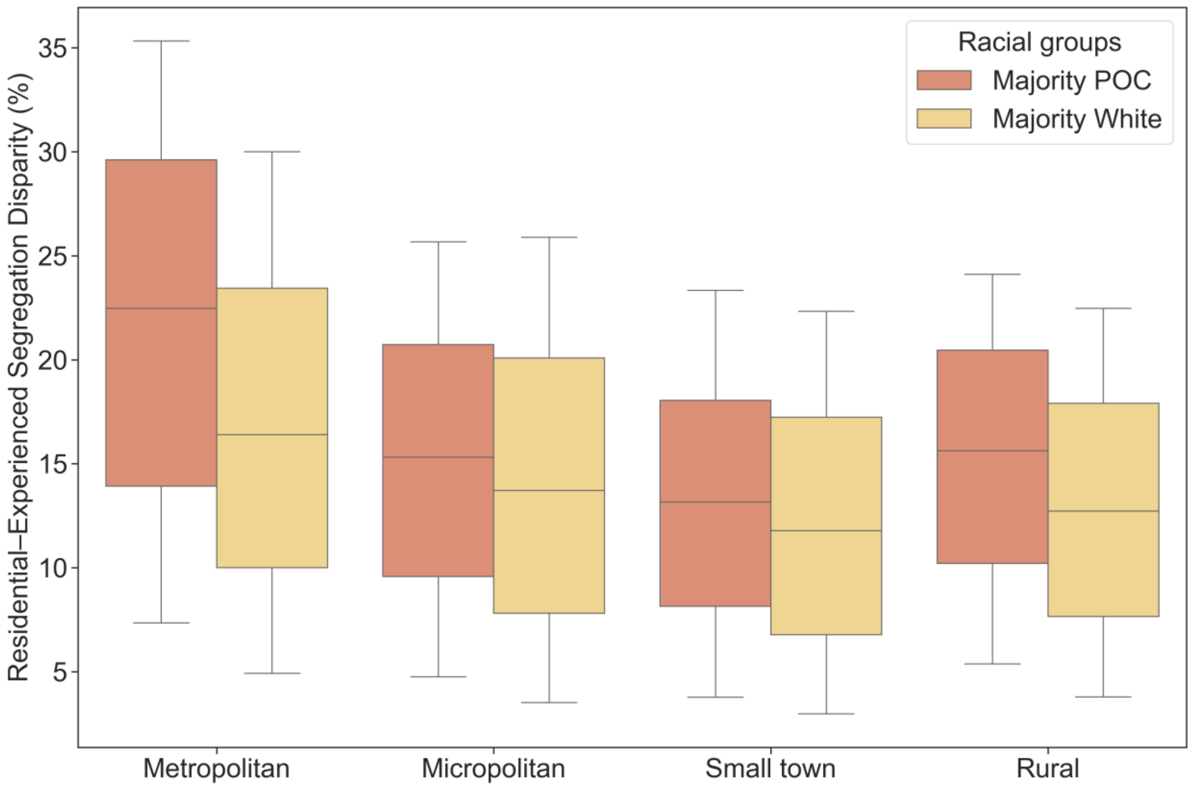

Supplementary Fig. 6 Residential-experienced segregation disparities between majority-POC (people of colour) and majority-White neighbourhoods across urbanicity levels. Disparity between residential and experienced segregation was calculated as (residential segregation – experienced segregation) / residential segregation. Only neighbourhoods where overall experienced segregation is lower than residential segregation were included in the calculation. In metropolitan areas, n=28,510 majority-POC neighbourhoods, and 102,768 majority-White neighbourhoods. In micropolitan areas, n=1,250 majority-POC neighbourhoods, and 13,655 majority-White neighbourhoods. In small towns, n=740 majority-POC neighbourhoods, and 7,036 majority-White neighbourhoods. In rural areas, n=423 majority-POC neighbourhoods, and 6,397 majority-White neighbourhoods. The box plots present the 10th, 25th, 50th, 75th, and 90th percentiles.

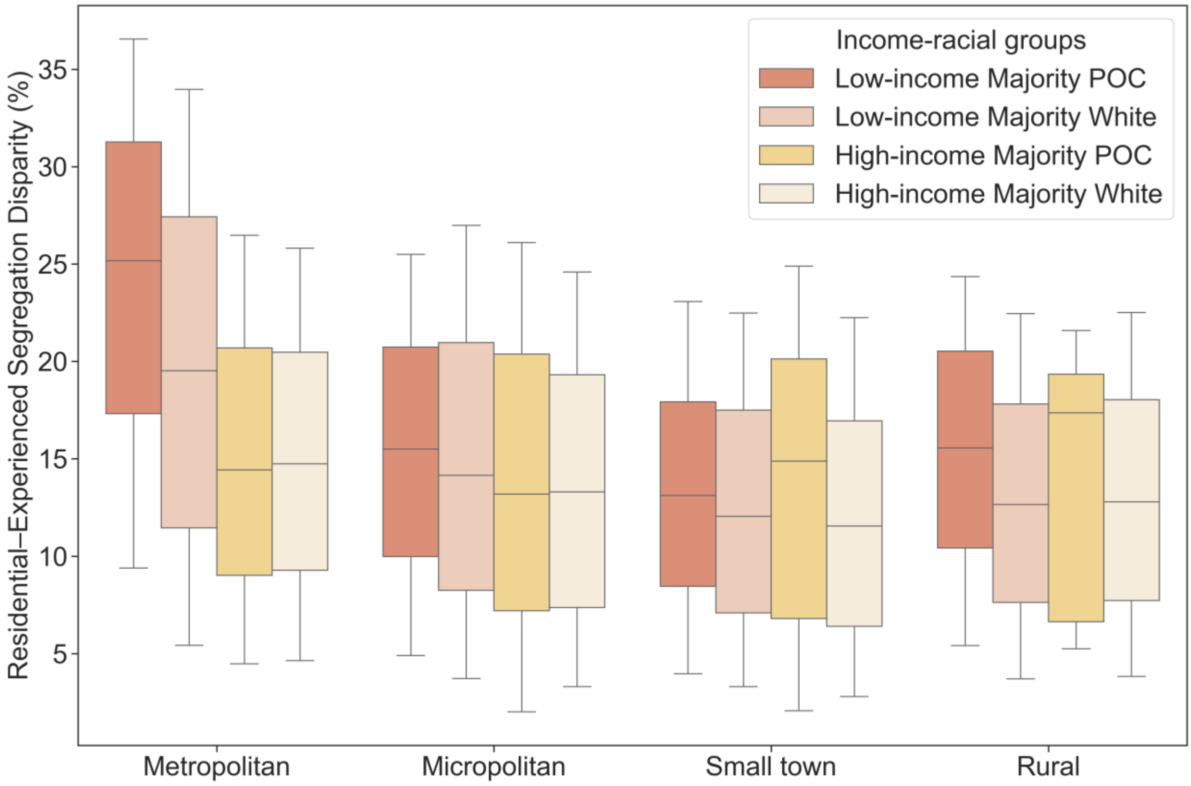

Supplementary Fig. 7 Residential-experienced segregation disparities between majority-POC (people of colour) and majority-White neighbourhoods across income-racial groups. Disparity between residential and experienced segregation was calculated as (residential segregation – experienced segregation) / residential segregation. Only neighbourhoods where overall experienced segregation is lower than residential segregation were included in the calculation. Neighbourhoods in the 1st and 2nd income quartiles were designated as low-income, and those in the 3rd and 4th quartiles as high-income. These were then cross-classified by racial composition into: (1) low-income, majority-POC (people of colour); (2) low-income, majority-White; (3) high-income, majority-POC; and (4) high-income, majority-White neighbourhoods. In metropolitan areas, n=21,498, 44,637, 7,012, and 58,131 neighbourhoods were classified as Low-income majority-POC, low-income majority-White, high-income majority POC, and high-income majority-White. In micropolitan areas, n=1,168, 6,996, 82, and 6,659 neighbourhoods were classified as the same groups. In small towns, n=676, 3,645, 64, and 3,391 neighbourhoods were classified as the same groups. In rural areas, n=391, 3,106, 32, and 3,291 neighbourhoods were classified as the same groups. The box plots present the 10th, 25th, 50th, 75th, and 90th percentiles.

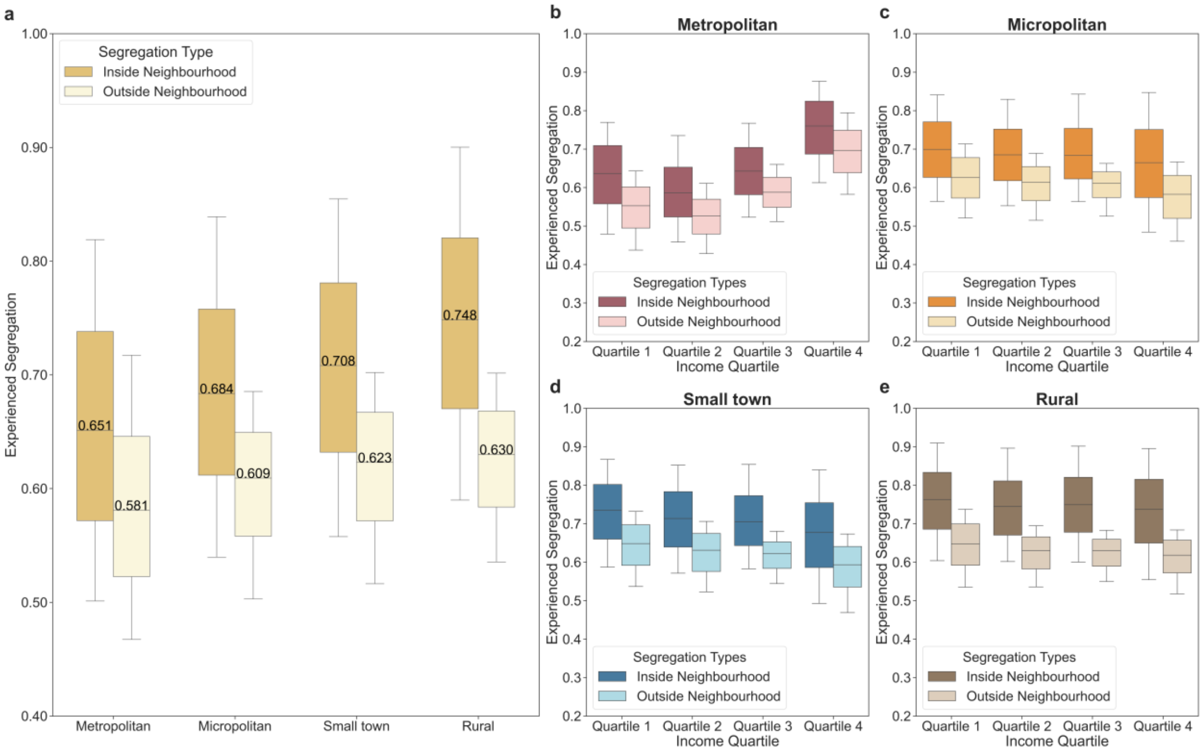

Supplementary Fig. 8 Experienced segregation for inside-neighbourhood and outside-neighbourhood trips across urbanicity levels and income groups. Noted that neighbourhoods with no record of inside-neighbourhood segregation or outside-neighbourhoods segregation were excluded in the analyses. In **a-e**, n=159,732, 19,907, 10,513, 8,286 neighbourhoods in metropolitan areas, micropolitan areas, small towns, and rural areas, respectively. The box plots in **a-e** present the 10th, 25th, 50th, 75th, and 90th percentiles.

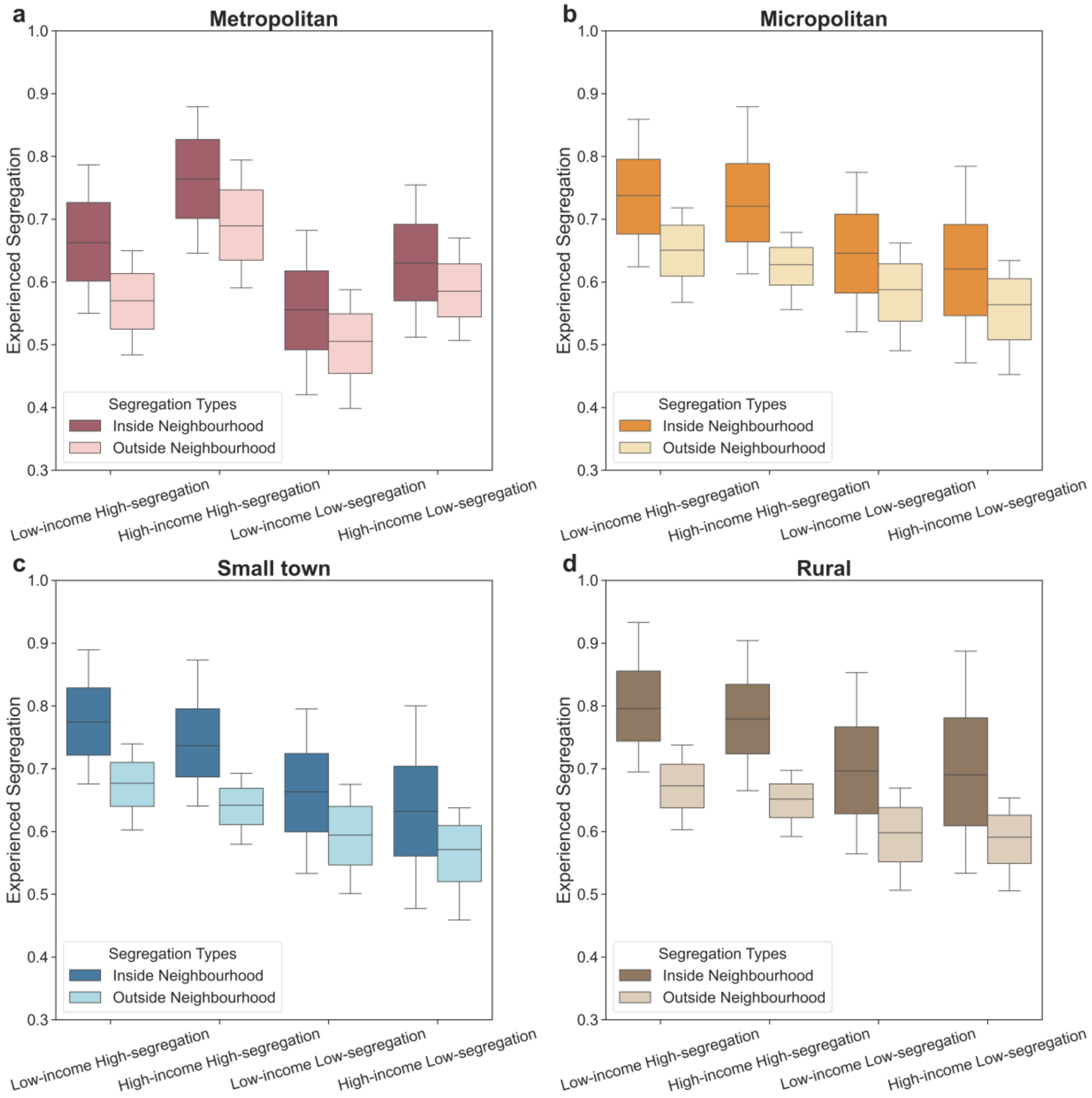

Supplementary Fig. 9 Experienced segregation for inside-neighbourhood and outside-neighbourhood trips by income and residential segregation levels across urbanicity levels. Noted that neighbourhoods with no record of inside-neighbourhood segregation or outside-neighbourhoods segregation were excluded in the analyses. In each urbanicity level, neighbourhoods in the 1st and 2nd income quartiles were classified as low-income group, and those in the 3rd and 4th quartiles as high-income group. Within each income group of each urbanicity level, neighbourhoods were further divided into low and high residential segregation groups based on the group-specific median. In metropolitan areas, n=39,933, 39,933, 39,933 and 39,933 neighbourhoods were classified as low-income high-segregation neighbourhoods, high-income high-segregation neighbourhoods, low-income low-segregation neighbourhoods, and high-income low-segregation neighbourhoods. In micropolitan areas, n=4,977, 4,976, 4,977, and 4,977 neighbourhoods were classified as the same groups. In small towns, n=2,628, 2,628, 2,629, and 2,628 neighbourhoods were classified as the same groups. In rural areas, n=2,071, 2,071, 2,072, and 2,072 neighbourhoods were classified as the same groups. The box plots present the 10th, 25th, 50th, 75th, and 90th percentiles.

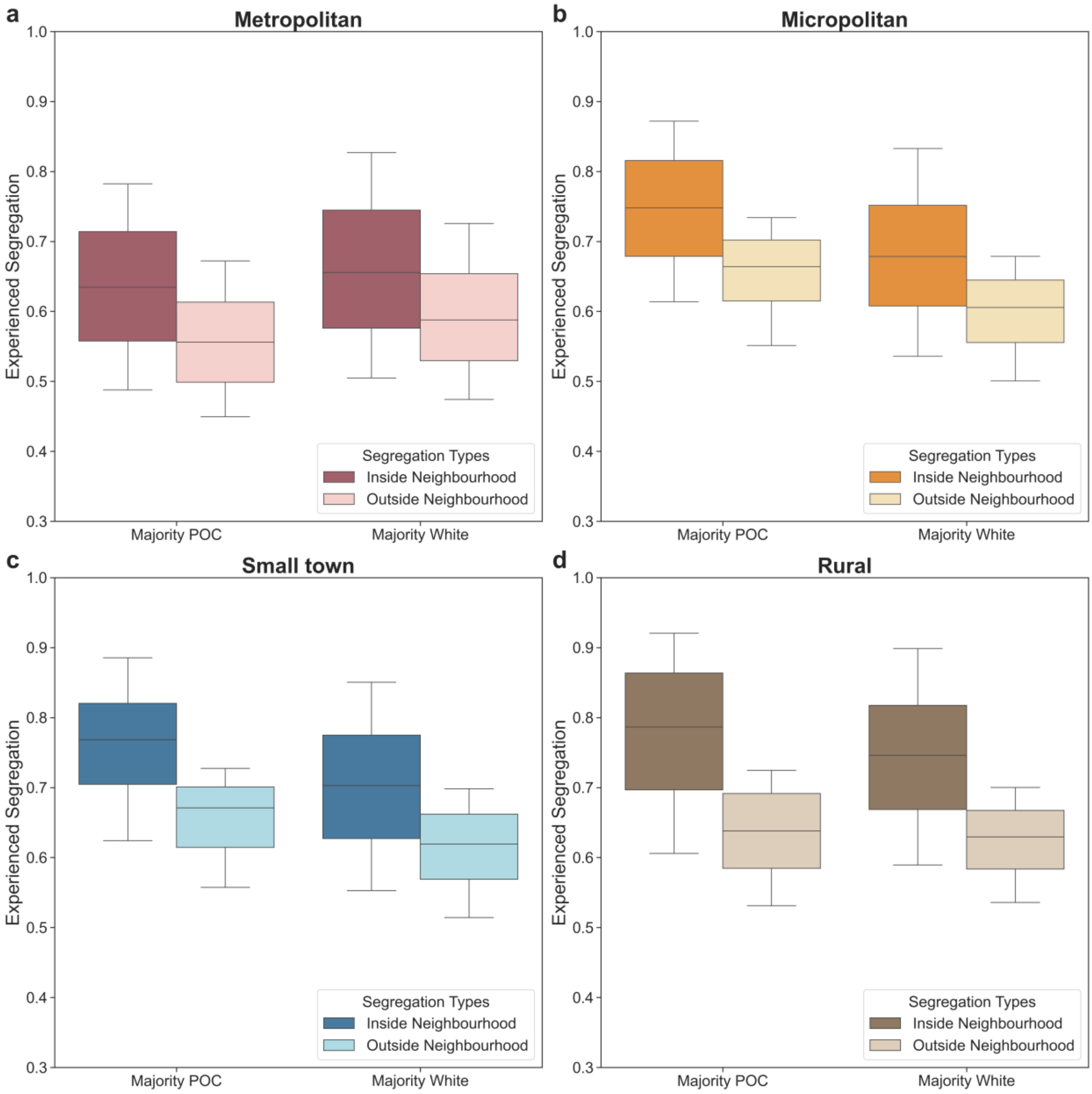

Supplementary Fig. 10 Experienced segregation for inside-neighbourhood and outside-neighbourhood trips between majority-POC (people of colour) and majority-White neighbourhoods across urbanicity levels. Noted that neighbourhoods with no record of inside-neighbourhood segregation or outside-neighbourhoods segregation were excluded in the analyses. In metropolitan areas, n=32,490 majority-POC neighbourhoods, and 127,242 majority-White neighbourhoods. In micropolitan areas, n=1,482 majority-POC neighbourhoods, and 18,425 majority-White neighbourhoods. In small towns, n=876 majority-POC neighbourhoods, and 9,637 majority-White neighbourhoods. In rural areas, n=435 majority-POC neighbourhoods, and 7,815 majority-White neighbourhoods. The box plots present the 10th, 25th, 50th, 75th, and 90th percentiles.

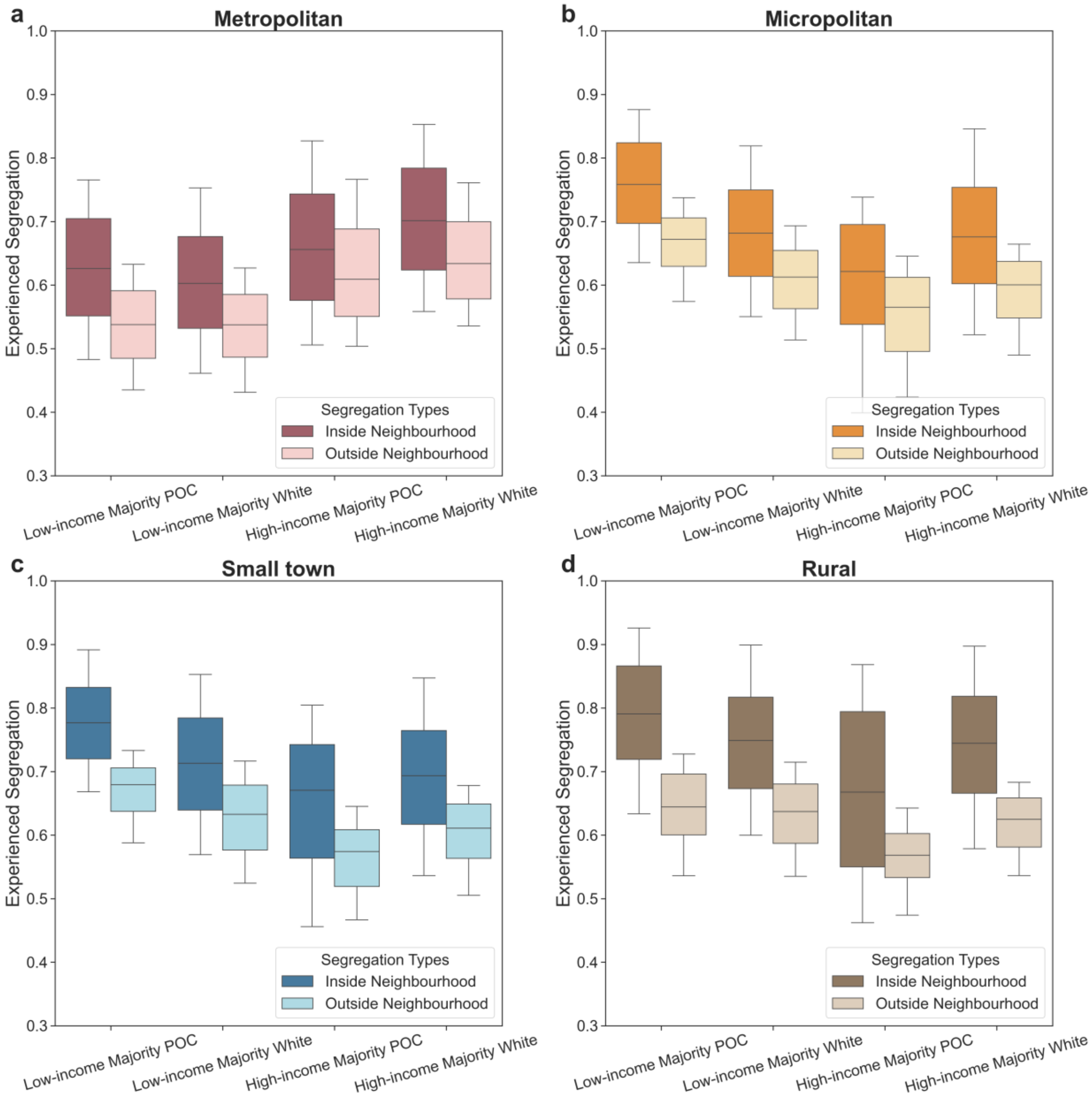

Supplementary Fig. 11 Experienced segregation for inside-neighbourhood and outside-neighbourhood trips by income-racial groups across urbanicity levels. Noted that neighbourhoods with no record of inside-neighbourhood segregation or outside-neighbourhoods segregation were excluded in the analyses. Neighbourhoods in the 1st and 2nd income quartiles were designated as low-income, and those in the 3rd and 4th quartiles as high-income. These were then cross-classified by racial composition into: (1) low-income, majority-POC (people of colour); (2) low-income, majority-White; (3) high-income, majority-POC; and (4) high-income, majority-White neighbourhoods. In metropolitan areas, n=23,401, 56,465, 9,089, and 70,777 neighbourhoods were classified as Low-income majority-POC, low-income majority-White, high-income majority-POC, and high-income majority-White. In micropolitan areas, n=1,338, 8,616, 144, and 9,809 neighbourhoods were classified as the same groups. In small towns, n=756, 4,501, 120, and 5,136 neighbourhoods were classified as the same groups. In rural areas, n=388, 3,755, 47, and 4,096 neighbourhoods were classified as the same groups. The box plots present the 10th, 25th, 50th, 75th, and 90th percentiles.

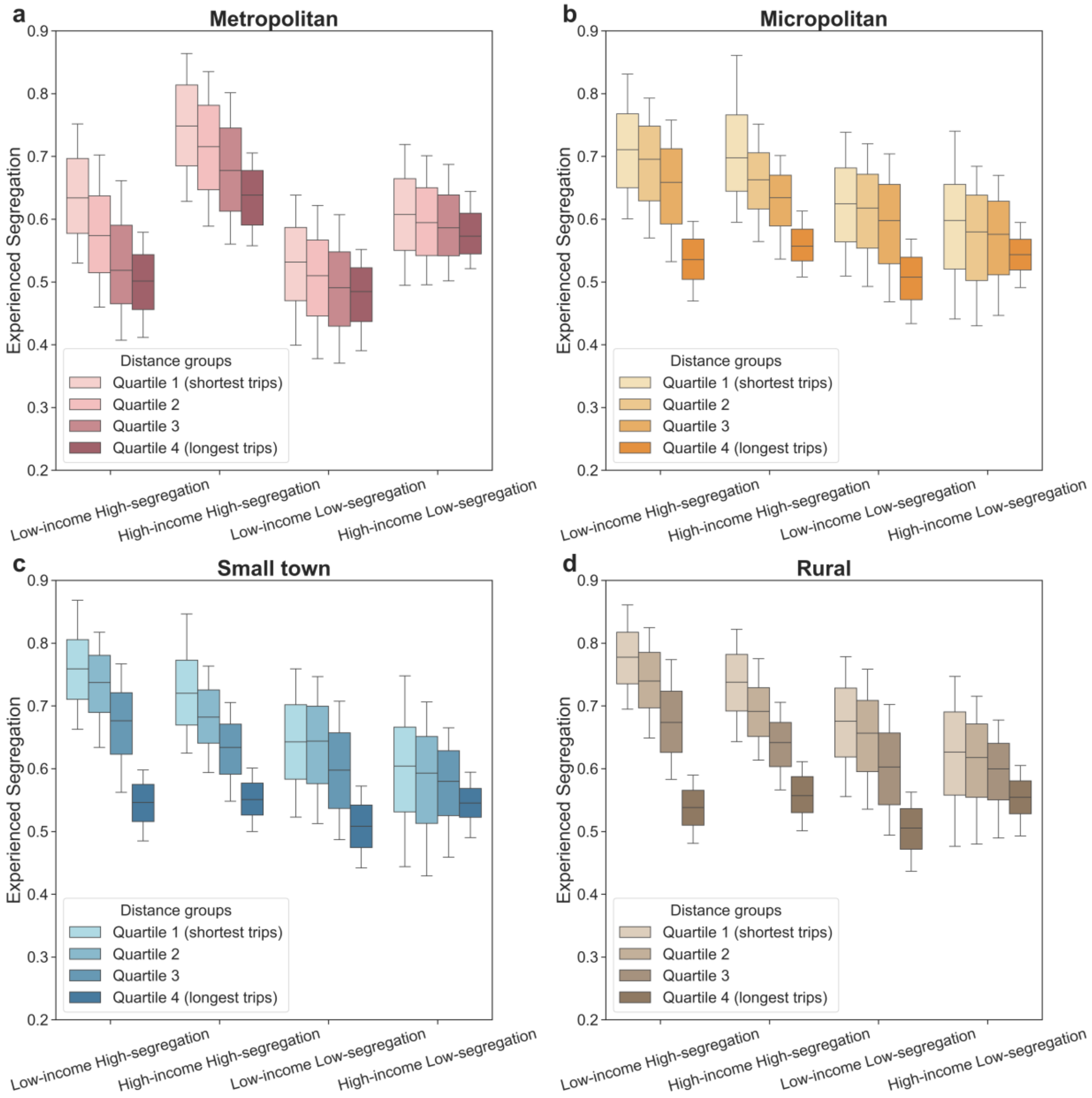

Supplementary Fig. 12 Patterns of experienced segregation decomposed by travel distance across neighbourhoods with different income and residential segregation levels and urbanicity levels. In each urbanicity level, neighbourhoods in the 1st and 2nd income quartiles were classified as low-income group, and those in the 3rd and 4th quartiles as high-income group. Within each income group of each urbanicity level, neighbourhoods were further divided into low and high residential segregation groups based on the group-specific median. In metropolitan areas, n=41,945, 41,945, 41,945 and 41,945 neighbourhoods were classified as low-income high-segregation neighbourhoods, high-income high-segregation neighbourhoods, low-income low-segregation neighbourhoods, and high-income low-segregation neighbourhoods. In micropolitan areas, n=5,145, 5,145, 5,145, and 5,145 neighbourhoods were classified as the same groups. In small towns, n=2,712, 2,711, 2,712, and 2,712 neighbourhoods were classified as the same groups. In rural areas, n=2,174, 2,167, 2,174, and 2,168 neighbourhoods were classified as the same groups. The box plots present the 10th, 25th, 50th, 75th, and 90th percentiles.

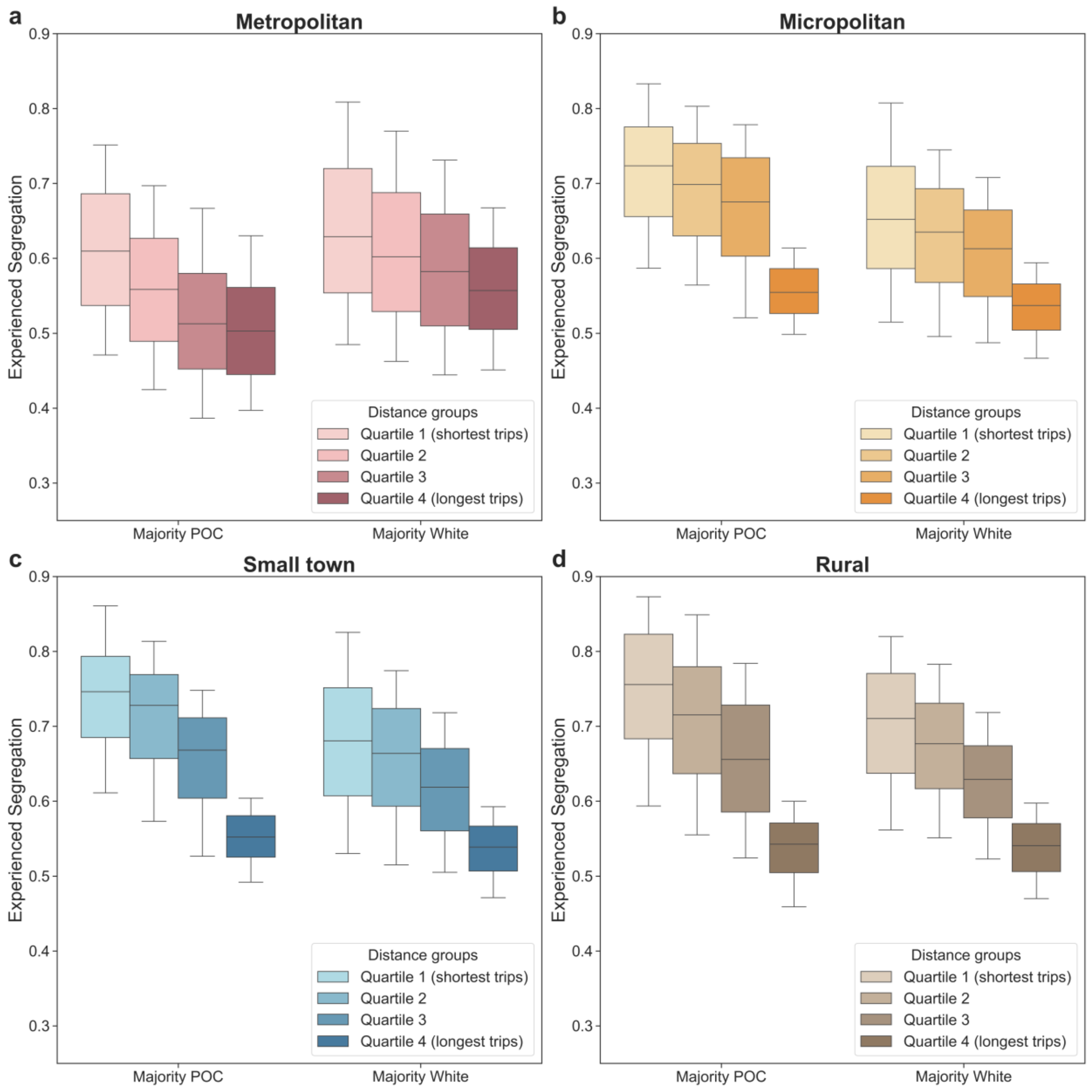

Supplementary Fig. 13 Patterns of experienced segregation decomposed by travel distance between majority-POC (people of colour) and majority-White neighbourhoods across urbanicity levels. In metropolitan areas, n=34,468 majority-POC neighbourhoods, and 133,312 majority-White neighbourhoods. In micropolitan areas, n=1,523 majority-POC neighbourhoods, and 19,057 majority-White neighbourhoods. In small towns, n=911 majority-POC neighbourhoods, and 9,936 majority-White neighbourhoods. In rural areas, n=489 majority-POC neighbourhoods, and 8,194 majority-White neighbourhoods. The box plots present the 10th, 25th, 50th, 75th, and 90th percentiles.

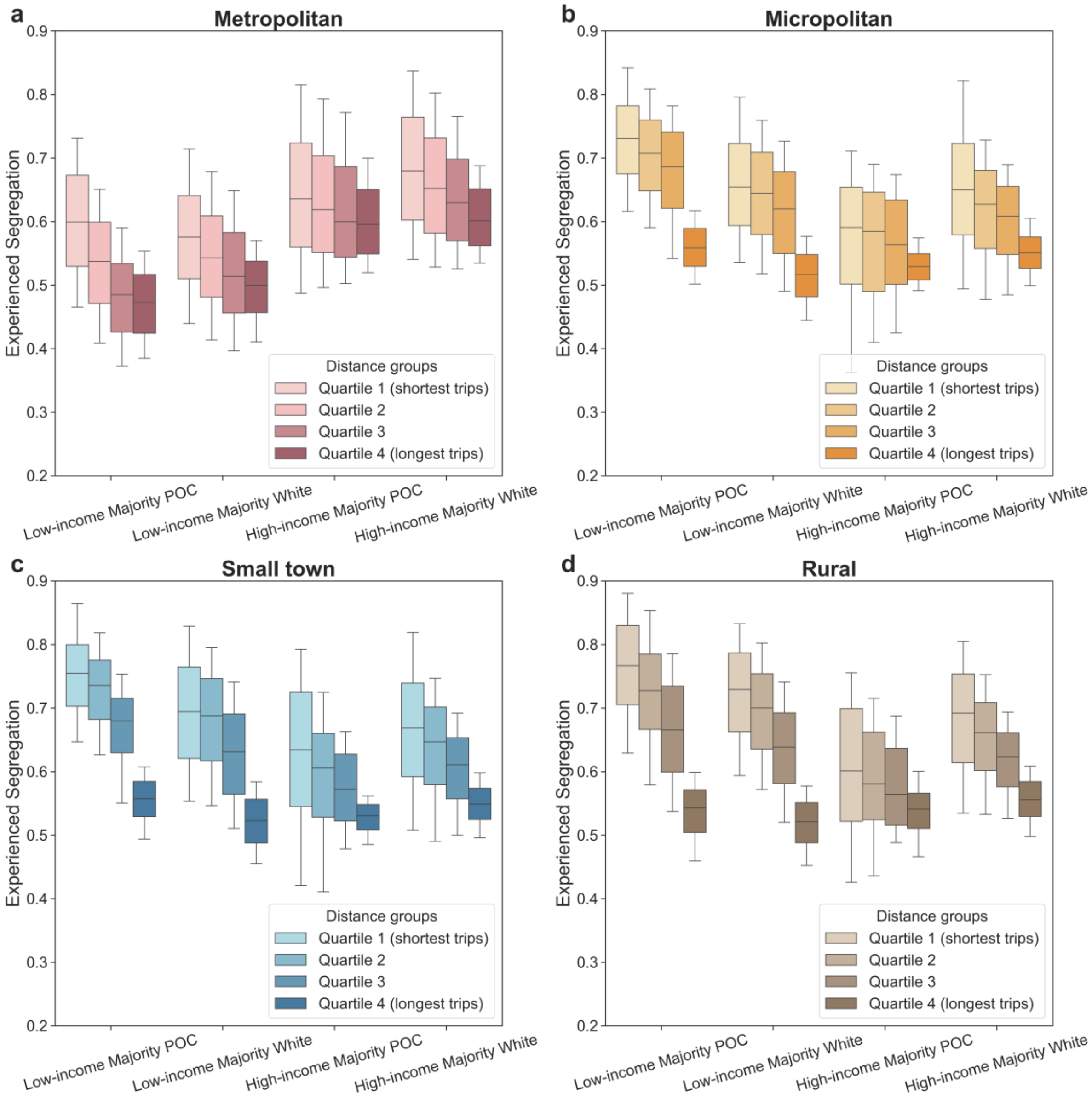

Supplementary Fig. 14 Patterns of experienced segregation decomposed by travel distance across income-racial groups and urbanicity levels. Neighbourhoods in the 1st and 2nd income quartiles were designated as low-income, and those in the 3rd and 4th quartiles as high-income. These were then cross-classified by racial composition into: (1) low-income, majority-POC (people of colour); (2) low-income, majority-White; (3) high-income, majority-POC; and (4) high-income, majority-White neighbourhoods. In metropolitan areas, n=24,749, 59,141, 9,719, and 74,171 neighbourhoods were classified as Low-income majority-POC, low-income majority-White, high-income majority-POC, and high-income majority-White. In micropolitan areas, n=1,371, 8,919, 152, and 10,138 neighbourhoods were classified as the same groups. In small towns, n=785, 4,639, 126, and 5,297 neighbourhoods were classified as the same groups. In rural areas, n=434, 3,914, 55, and 4,280 neighbourhoods were classified as the same groups. The box plots present the 10th, 25th, 50th, 75th, and 90th percentiles.

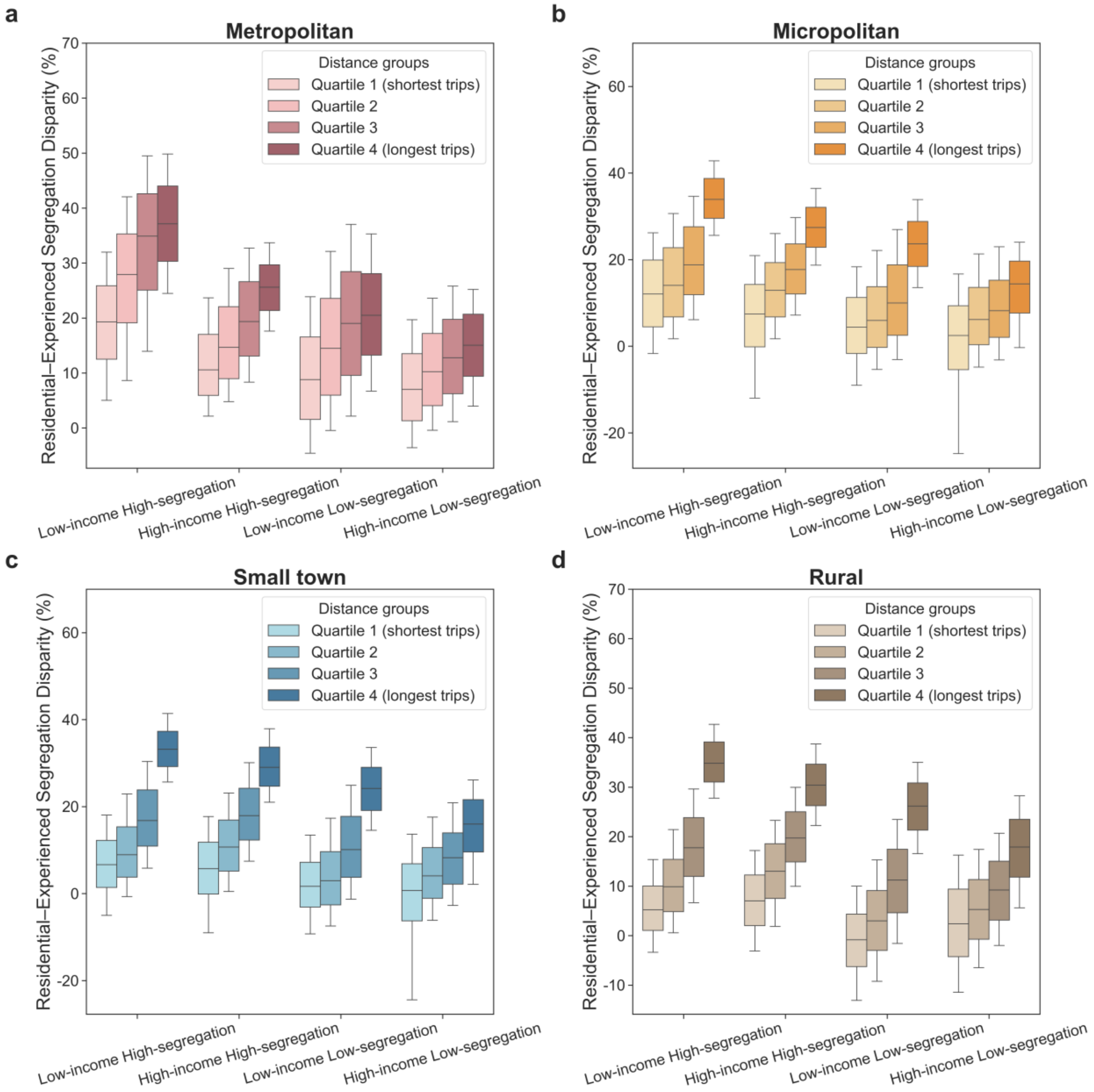

Supplementary Fig. 15 Disparities between residential segregation and experienced segregation decomposed by travel distance quartiles across income-residential levels and urbanicity levels. Disparity was calculated as (residential segregation – experienced segregation) / residential segregation. Only neighbourhoods where overall experienced segregation is lower than residential segregation were included in the calculation. In each urbanicity level, neighbourhoods in the 1st and 2nd income quartiles were classified as low-income group, and those in the 3rd and 4th quartiles as high-income group. Within each income group of each urbanicity level, neighbourhoods were further divided into low and high residential segregation groups based on the group-specific median. In metropolitan areas, n=33,072, 32,566, 33,073 and 32,567 neighbourhoods were classified as low-income high-segregation neighbourhoods, high-income high-segregation neighbourhoods, low-income low-segregation neighbourhoods, and high-income low-segregation neighbourhoods. In micropolitan areas, n=4,084, 3,368, 4,084, and 3,369 neighbourhoods were classified as the same groups. In small towns, n=2,162, 1,725, 2,163, and 1,726 neighbourhoods were classified as the same groups. In rural areas, n=1,759, 1,651, 1,759, and 1,651 neighbourhoods were classified as the same groups. The box plots present the 10th, 25th, 50th, 75th, and 90th percentiles.

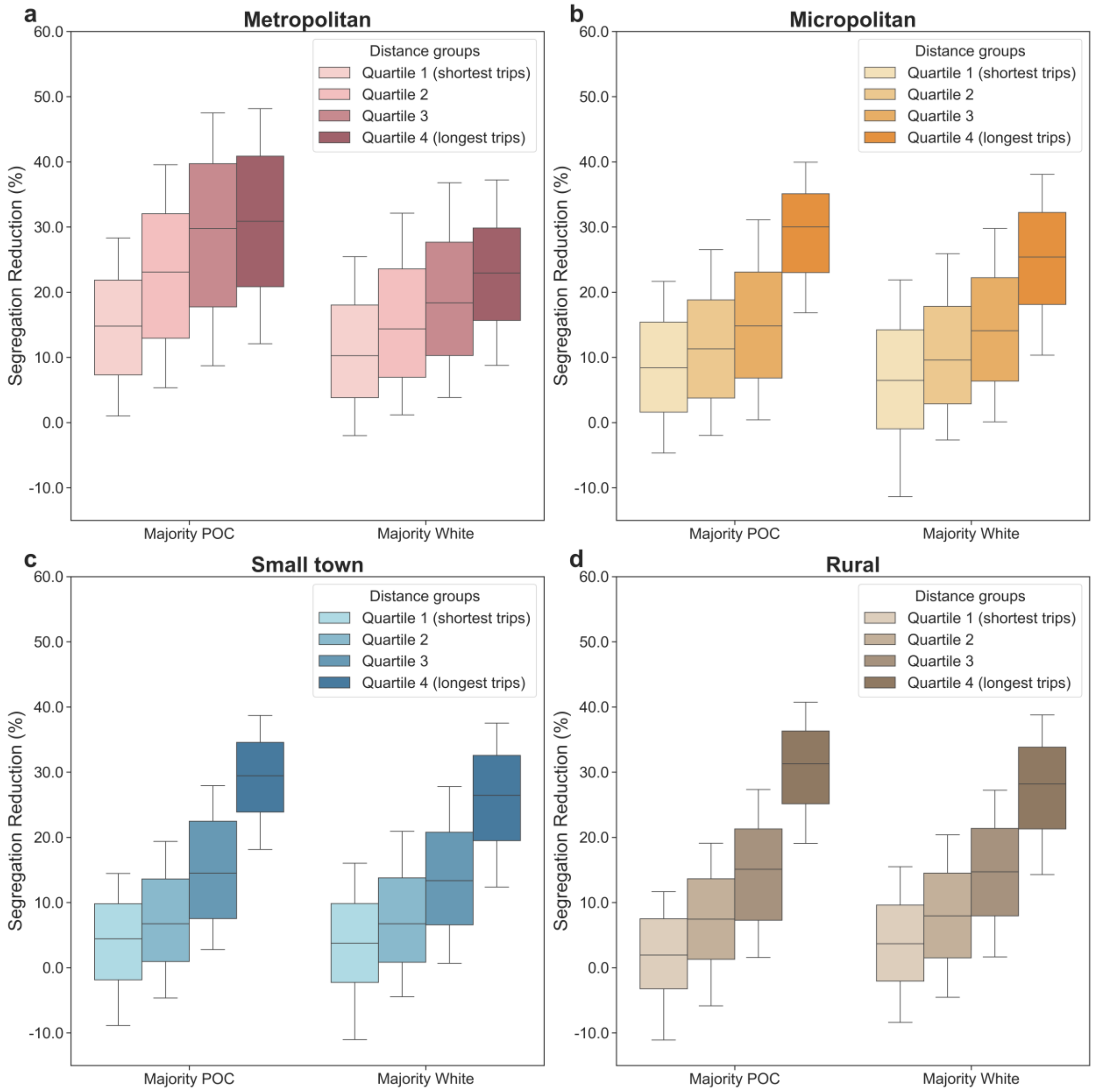

Supplementary Fig. 16 Disparities between residential segregation and experienced segregation decomposed by travel distance quartiles between majority-POC (people of colour) and majority-White neighbourhoods across urbanicity levels. Disparity was calculated as (residential segregation – experienced segregation) / residential segregation. Only neighbourhoods where overall experienced segregation is lower than residential segregation were included in the calculation. In metropolitan areas, n=28,510 majority-POC neighbourhoods, and 102,768 majority-White neighbourhoods. In micropolitan areas, n=1,250 majority-POC neighbourhoods, and 13,655 majority-White neighbourhoods. In small towns, n=740 majority-POC neighbourhoods, and 7,036 majority-White neighbourhoods. In rural areas, n=423 majority-POC neighbourhoods, and 6,397 majority-White neighbourhoods. The box plots present the 10th, 25th, 50th, 75th, and 90th percentiles.

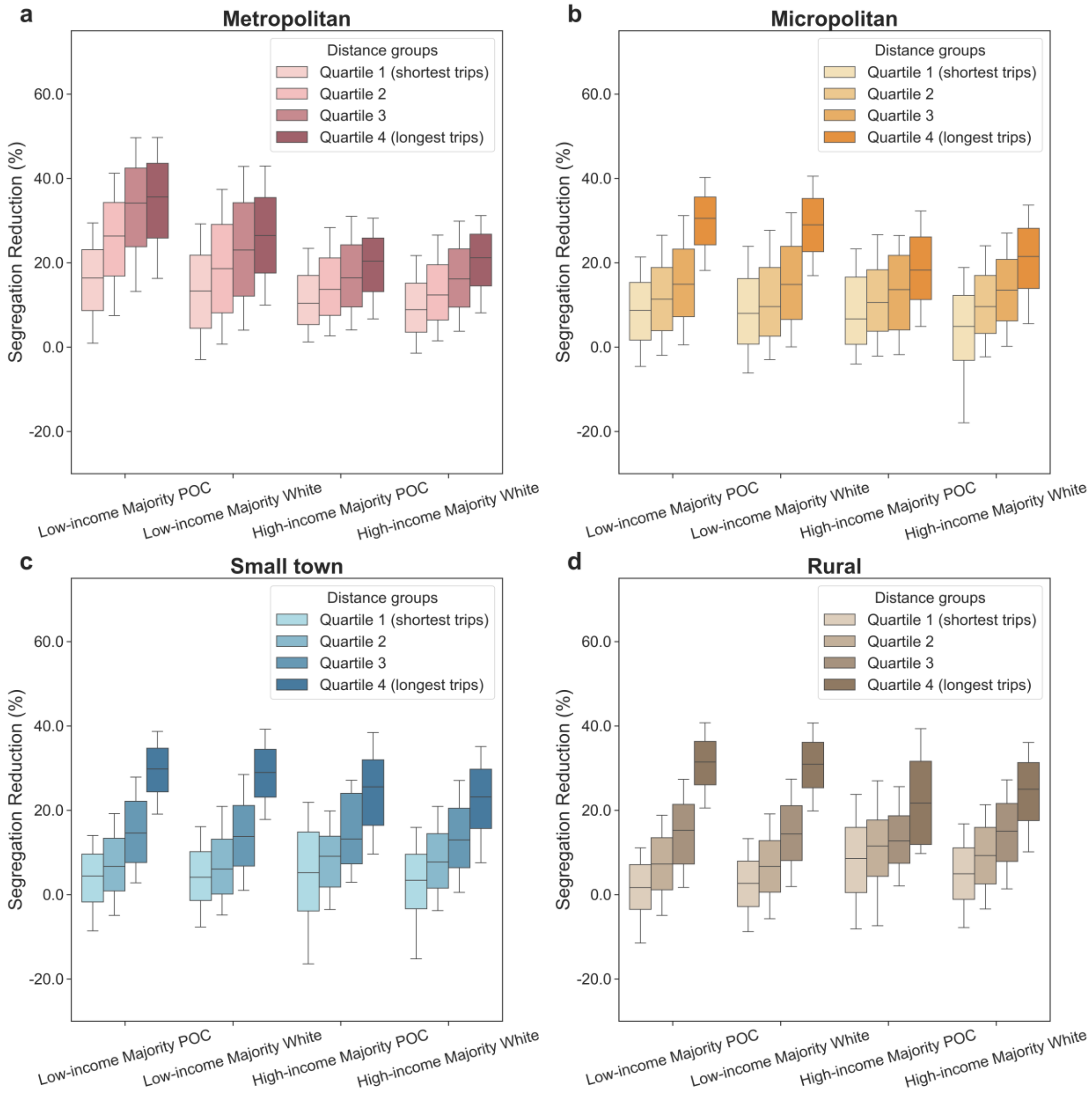

Supplementary Fig. 17 Disparities between residential segregation and experienced segregation decomposed by travel distance quartiles across income-racial groups and urbanicity levels. Disparity was calculated as (residential segregation – experienced segregation) / residential segregation. Only neighbourhoods where overall experienced segregation is lower than residential segregation were included in the calculation. Neighbourhoods in the 1st and 2nd income quartiles were designated as low-income, and those in the 3rd and 4th quartiles as high-income. These were then cross-classified by racial composition into: (1) low-income, majority-POC (people of colour); (2) low-income, majority-White; (3) high-income, majority-POC; and (4) high-income, majority-White neighbourhoods. In metropolitan areas, n=21,498, 44,637, 7,012, and 58,131 neighbourhoods were classified as Low-income majority-POC, low-income majority-White, high-income majority-POC, and high-income majority-White. In micropolitan areas, n=1,168, 6,996, 82, and 6,659 neighbourhoods were classified as the same groups. In small towns, n=676, 3,645, 64, and 3,391 neighbourhoods were classified as the same groups. In rural areas, n=391, 3,106, 32, and 3,291 neighbourhoods were classified as the same groups. The box plots present the 10th, 25th, 50th, 75th, and 90th percentiles.

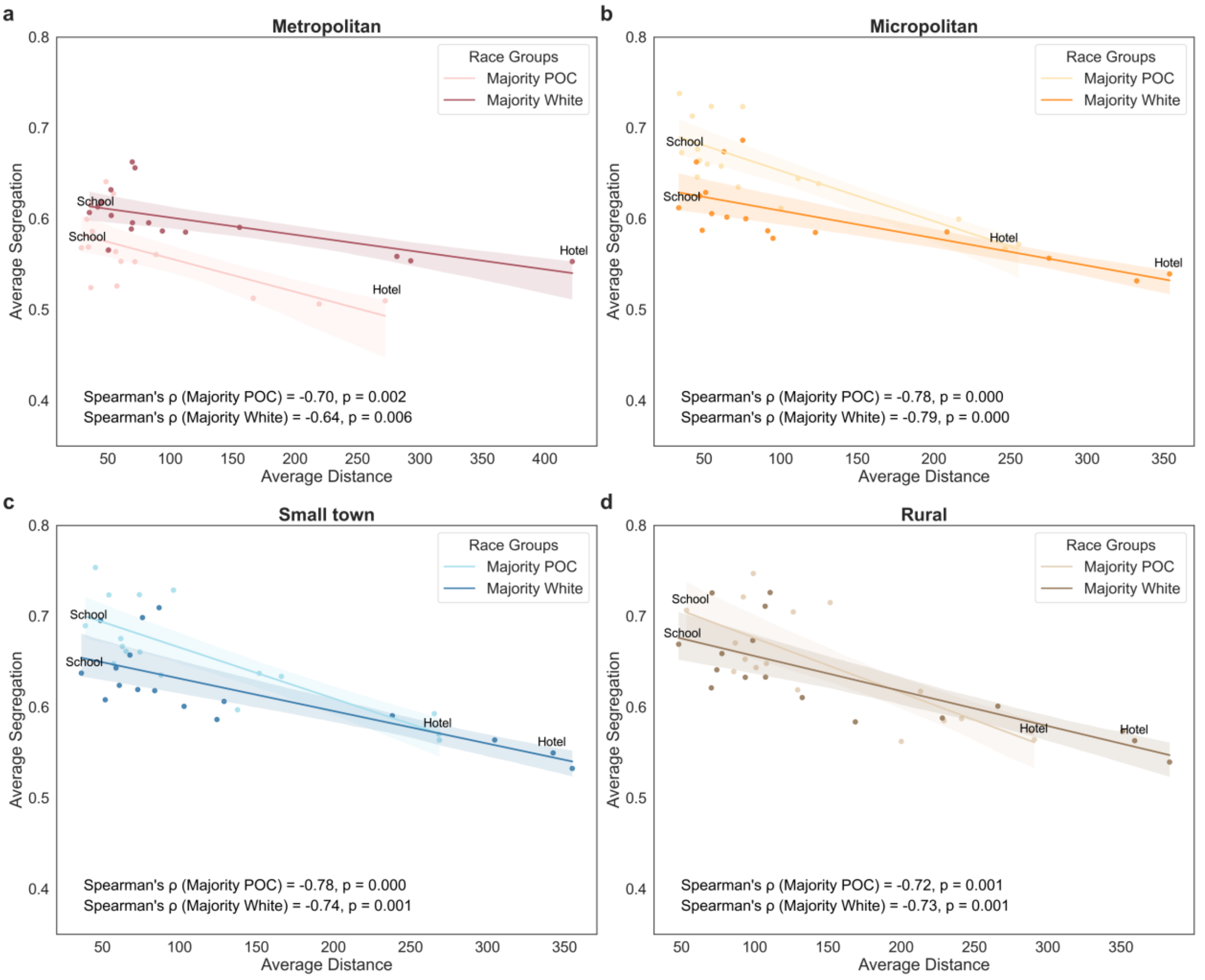

Supplementary Fig. 18 Associations between average experienced segregation and average travel distance at the level of activity sites between majority-POC (people of colour) and majority-White neighbourhoods across urbanicity levels. In **a-d**, The texts show results of spearman correlations between experienced segregation and travel distance for activity sites. Statistical significance was assessed using two-sided tests. Each dot represents a specific type of activity site within an income quartile, with n = 16 types. Lines represent linear associations for each income quartile, with shaded bands indicating 95% confidence intervals.

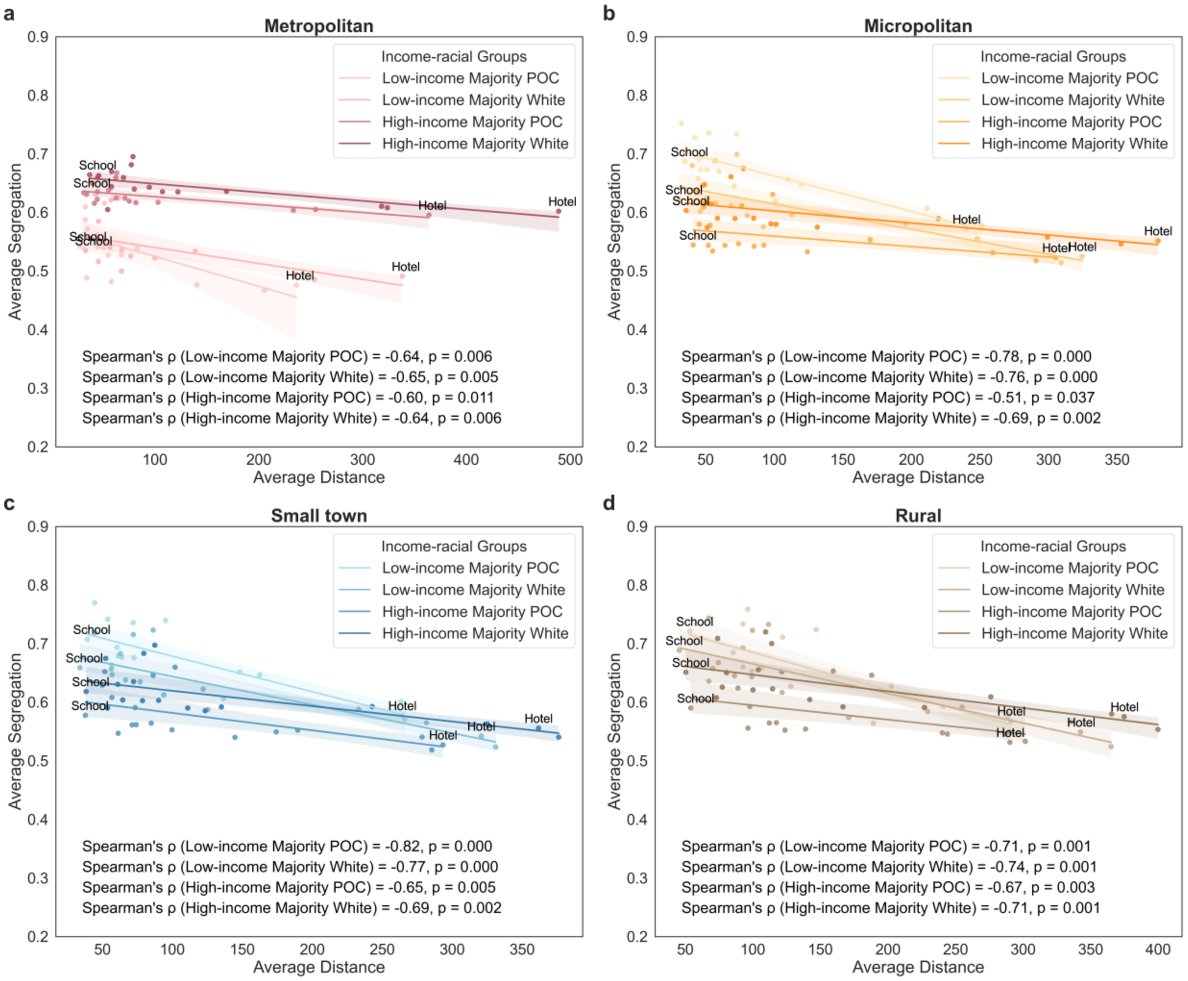

Supplementary Fig. 19 Associations between average experienced segregation and average travel distance at the level of activity sites across income-racial groups and urbanicity levels. In **a-d**, The texts show results of spearman correlations between experienced segregation and travel distance for activity sites. Statistical significance was assessed using two-sided tests. Each dot represents a specific type of activity site within an income quartile, with n = 16 types. Lines represent linear associations for each income quartile, with shaded bands indicating 95% confidence intervals.

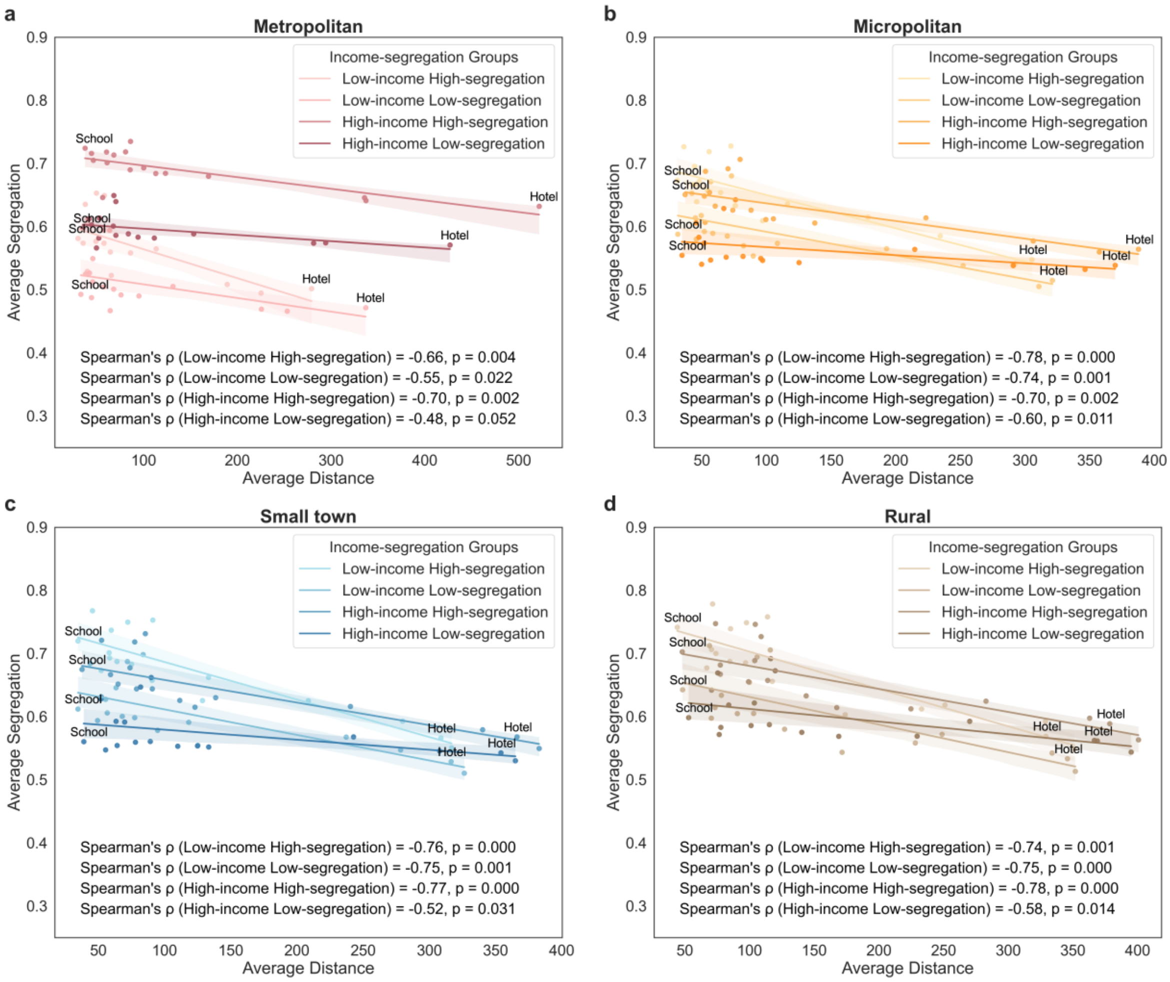

Supplementary Fig. 20 Associations between average experienced segregation and average travel distance at the level of activity sites by income and residential segregation levels across urbanicity levels. In **a-d**, The texts show results of spearman correlations between experienced segregation and travel distance for activity sites. Statistical significance was assessed using two-sided tests. Each dot represents a specific type of activity site within an income quartile, with $n = 16$ types. Lines represent linear associations for each income quartile, with shaded bands indicating 95% confidence intervals.

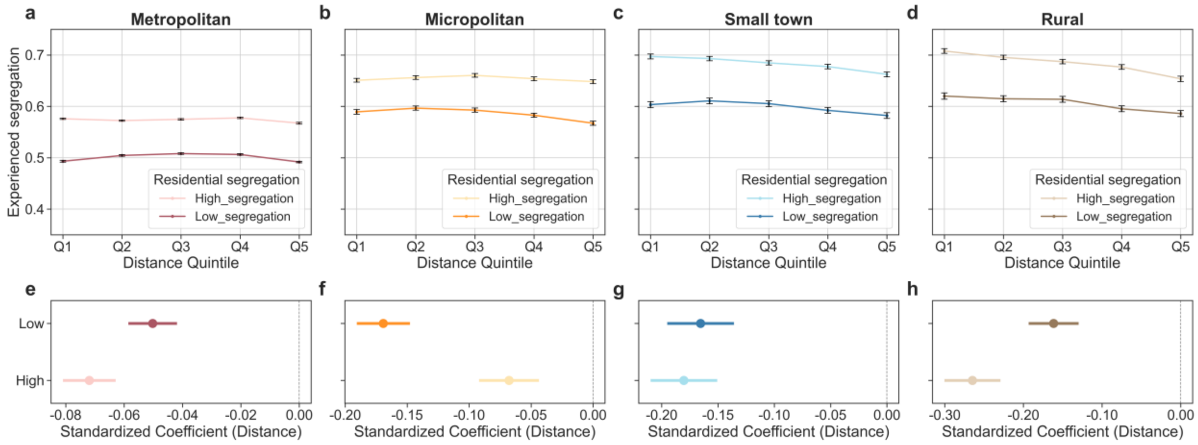

Supplementary Fig. 21 Associations between average travel distance and experienced segregation between low-income neighbourhoods with high and low residential segregation. a-d. Experienced segregation by average travel distance between low-income neighbourhoods with high and low residential segregation across urbanicity levels. In **a-d**, Q1, Q2, Q3, Q4, and Q5 denote the 1st, 2nd, 3rd, 4th, and 5th travel distance quintiles, respectively. For metropolitan areas, n=41,945 and 41,945 neighbourhoods with high and low residential segregation, respectively. For micropolitan areas, n=5,145 and 5,145 neighbourhoods with high and low residential segregation, respectively. For small towns, n=2,712 and 2,712 neighbourhoods with high and low residential segregation, respectively. For rural areas, n=2,174 and 2,174 neighbourhoods with high and low residential segregation, respectively. Points show the mean experienced segregation for each group across travel distance quintiles within each urbanicity level, with error bars indicating 95% confidence intervals. **e-h.** The coefficients of travel distance for each group across urbanicity levels from multiple linear regression models (n=83, 890 neighbourhoods for metropolitan areas, n=10,290 neighbourhoods for micropolitan areas, n=5,424 neighbourhoods for small towns, and n=4,348 neighbourhoods for rural areas). The error bars indicate 95% confidence intervals.

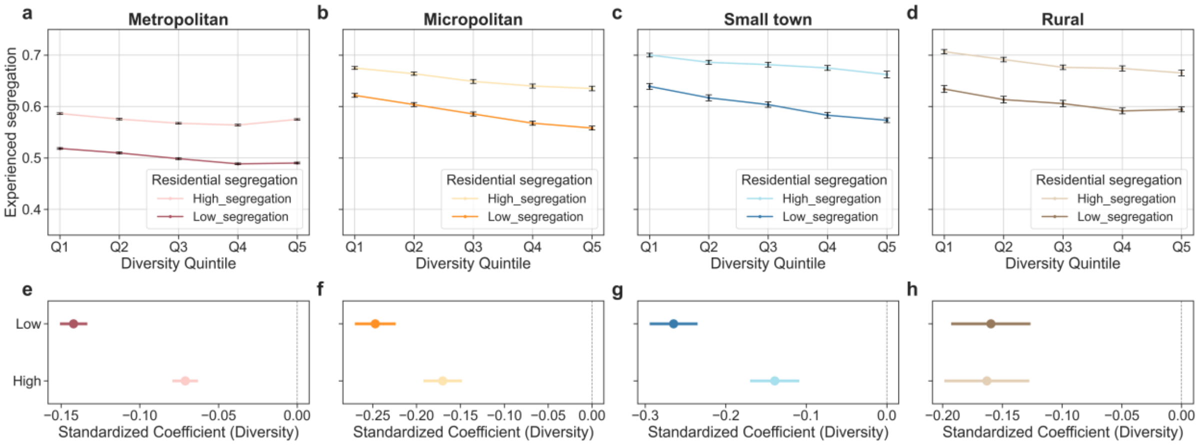

Supplementary Fig. 22 Associations between travel diversity and experienced segregation between low-income neighbourhoods with high and low residential segregation. a-d. Experienced segregation by travel diversity between low-income neighbourhoods with high and low residential segregation across urbanicity levels. In **a-d**, Q1, Q2, Q3, Q4, and Q5 denote the 1st, 2nd, 3rd, 4th, and 5th travel diversity quintiles, respectively. For metropolitan areas, n=41,945 and 41,945 neighbourhoods with high and low residential segregation, respectively. For micropolitan areas, n=5,145 and 5,145 neighbourhoods with high and low residential segregation, respectively. For small towns, n=2,712 and 2,712 neighbourhoods with high and low residential segregation, respectively. For rural areas, n=2,174 and 2,174 neighbourhoods with high and low residential segregation, respectively. Points show the mean experienced segregation for each group across travel diversity quintiles within each urbanicity level, with error bars indicating 95% confidence intervals. **e-h.** The coefficients of travel diversity for each group across urbanicity levels from multiple linear regression models (n=83, 890 neighbourhoods for metropolitan areas, n=10,290 neighbourhoods for micropolitan areas, n=5,424 neighbourhoods for small towns, and n=4,348 neighbourhoods for rural areas). The error bars indicate 95% confidence intervals.

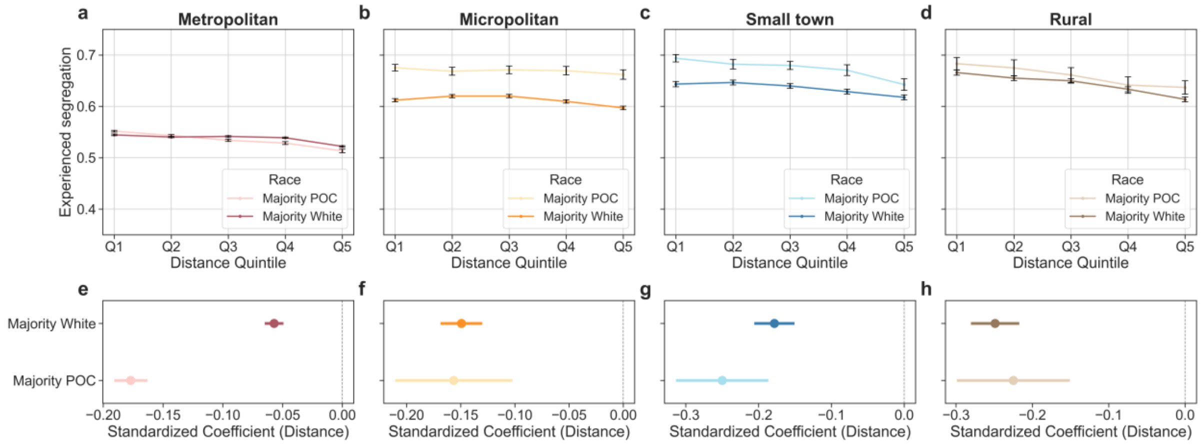

Supplementary Fig. 23 Associations between average travel distance and experienced segregation across low-income neighbourhoods with majority-POC (people of colour) and majority-White. a-d. Experienced segregation by average travel distance between low-income neighbourhoods with majority-POC and majority-White across urbanicity levels. In **a-d**, Q1, Q2, Q3, Q4, and Q5 denote the 1st, 2nd, 3rd, 4th, and 5th travel distance quintiles, respectively. For metropolitan areas, n=24,749 majority-POC neighbourhoods and 59,141 majority-White neighbourhoods, respectively. For micropolitan areas, n=1,371 majority-POC neighbourhoods and 8,919 majority-White neighbourhoods, respectively. For small towns, n=785 majority-POC neighbourhoods and 4,639 majority-White neighbourhoods, respectively. For metropolitan areas, n=434 majority-POC neighbourhoods and 3,914 majority-White neighbourhoods, respectively. Points show the mean experienced segregation for each group across travel distance quintiles within each urbanicity level, with error bars indicating 95% confidence intervals. **e-h.** The coefficients of travel distance for each group across urbanicity levels from multiple linear regression models (n=83, 890 neighbourhoods for metropolitan areas, n=10,290 neighbourhoods for micropolitan areas, n=5,424 neighbourhoods for small towns, and n=4,348 neighbourhoods for rural areas). The error bars indicate 95% confidence intervals.

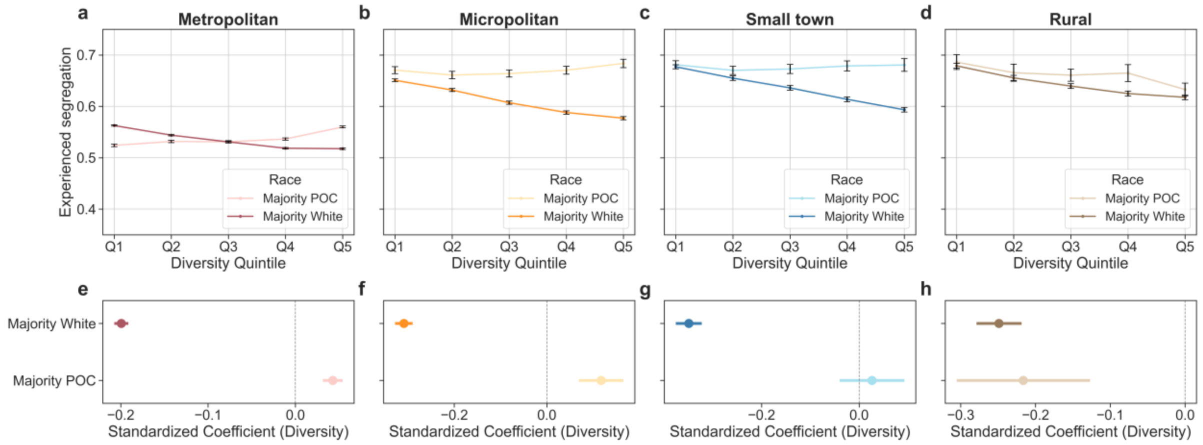

Supplementary Fig. 24 Associations between travel diversity and experienced segregation across low-income neighbourhoods with majority-POC (people of colour) and majority-White. a-d. Experienced segregation by travel diversity between low-income neighbourhoods with majority-POC and majority-White across urbanicity levels. In **a-d**, Q1, Q2, Q3, Q4, and Q5 denote the 1st, 2nd, 3rd, 4th, and 5th travel diversity quintiles, respectively. For metropolitan areas, n=24,749 majority-POC neighbourhoods and 59,141 majority-White neighbourhoods, respectively. For micropolitan areas, n=1,371 majority-POC neighbourhoods and 8,919 majority-White neighbourhoods, respectively. For small towns, n=785 majority-POC neighbourhoods and 4,639 majority-White neighbourhoods, respectively. For metropolitan areas, n=434 majority-POC neighbourhoods and 3,914 majority-White neighbourhoods, respectively. Points show the mean experienced segregation for each group across travel diversity quintiles within each urbanicity level, with error bars indicating 95% confidence intervals. **e-h.** The coefficients of travel diversity for each group across urbanicity levels from multiple linear regression models (n=83, 890 neighbourhoods for metropolitan areas, n=10,290 neighbourhoods for micropolitan areas, n=5,424 neighbourhoods for small towns, and n=4,348 neighbourhoods for rural areas). The error bars indicate 95% confidence intervals.

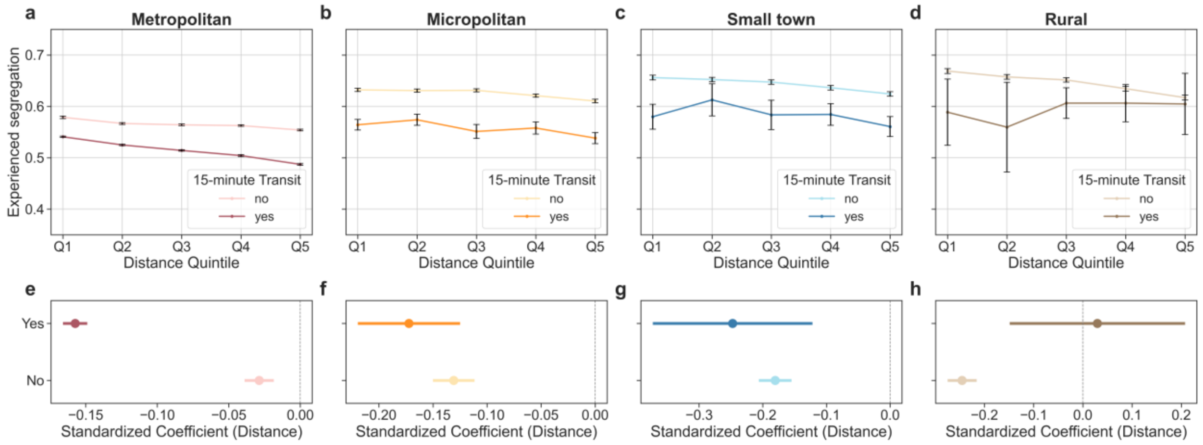

Supplementary Fig. 25 Associations between average travel distance and experienced segregation across low-income neighbourhoods with and without public transit stops within a 15-minute walking distance. a-d. Experienced segregation by average travel distance between low-income neighbourhoods with and without 15-minute public transits across urbanicity levels. In **a-d**, Q1, Q2, Q3, Q4, and Q5 denote the 1st, 2nd, 3rd, 4th, and 5th travel distance quintiles, respectively. For metropolitan areas, n=47,469 and 36,412 neighbourhoods with and without 15-minute public transits, respectively. For micropolitan areas, n=850 and 9,440 neighbourhoods with and without 15-minute public transits, respectively. For small towns, n=203 and 5,221 neighbourhoods with and without 15-minute public transits, respectively. For rural areas, n=56 and 4,292 neighbourhoods with and without 15-minute public transits, respectively. Points show the mean experienced segregation for each group across travel distance quintiles within each urbanicity level, with error bars indicating 95% confidence intervals. **e-h.** The coefficients of travel distance for each group across urbanicity levels from multiple linear regression models (n=83, 890 neighbourhoods for metropolitan areas, n=10,290 neighbourhoods for micropolitan areas, n=5,424 neighbourhoods for small towns, and n=4,348 neighbourhoods for rural areas). The error bars indicate 95% confidence intervals.

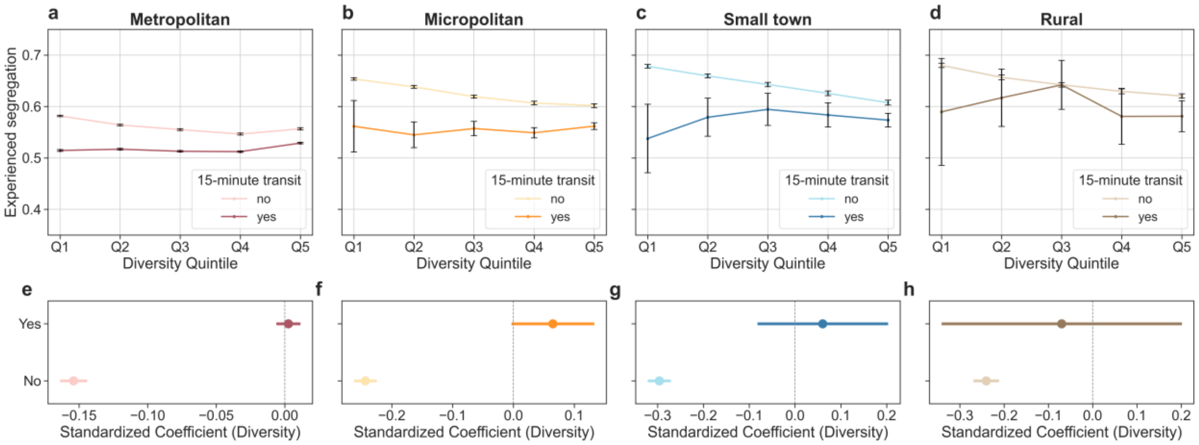

Supplementary Fig. 26 Associations between travel diversity and experienced segregation across low-income neighbourhoods with and without public transit stops within a 15-minute walking distance. a-d. Experienced segregation by travel diversity between low-income neighbourhoods with and without 15-minute public transits across urbanicity levels. In **a-d**, Q1, Q2, Q3, Q4, and Q5 denote the 1st, 2nd, 3rd, 4th, and 5th travel diversity quintiles, respectively. For metropolitan areas, n=47,469 and 36,412 neighbourhoods with and without 15-minute public transits, respectively. For micropolitan areas, n=850 and 9,440 neighbourhoods with and without 15-minute public transits, respectively. For small towns, n=203 and 5,221 neighbourhoods with and without 15-minute public transits, respectively. For rural areas, n=56 and 4,292 neighbourhoods with and without 15-minute public transits, respectively. Points show the mean experienced segregation for each group across travel diversity quintiles within each urbanicity level, with error bars indicating 95% confidence intervals. **e-h.** The coefficients of travel diversity for each group across urbanicity levels from multiple linear regression models (n=83, 890 neighbourhoods for metropolitan areas, n=10,290 neighbourhoods for micropolitan areas, n=5,424 neighbourhoods for small towns, and n=4,348 neighbourhoods for rural areas). The error bars indicate 95% confidence intervals.

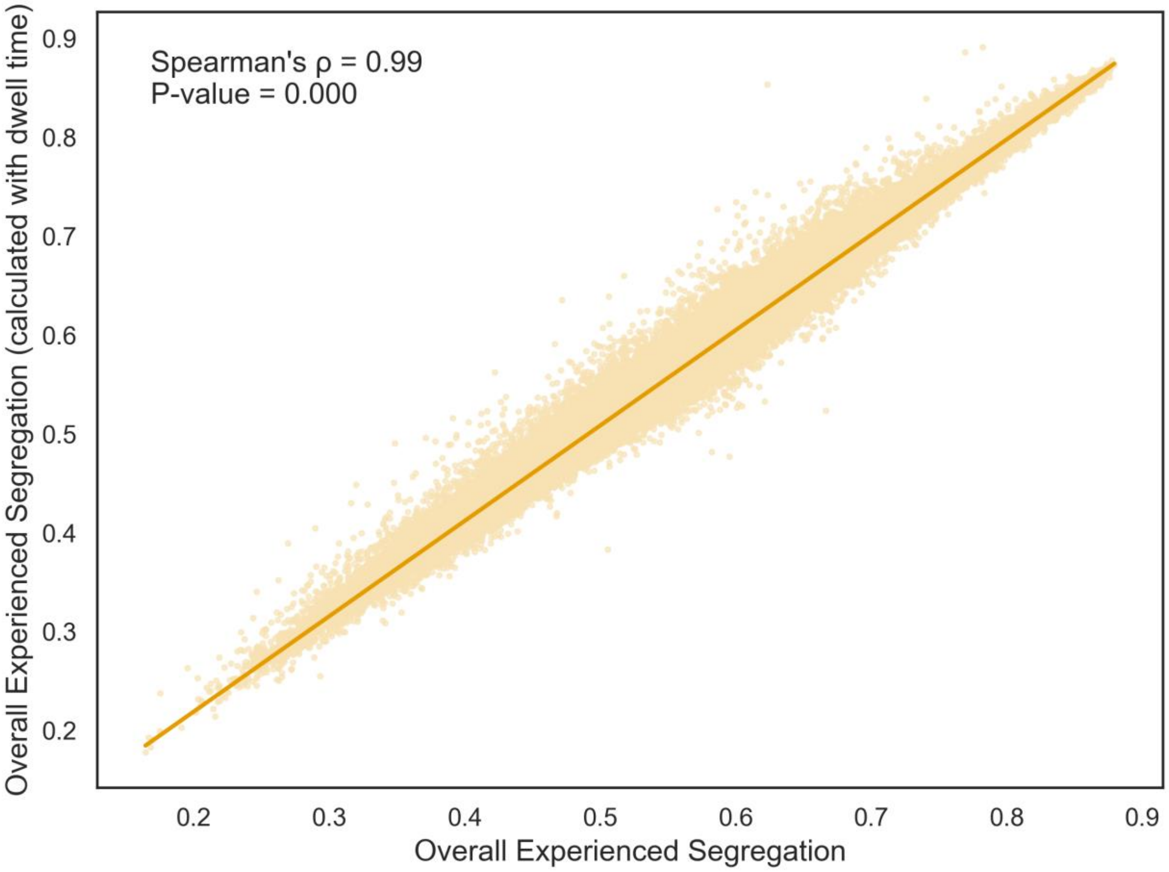

Supplementary Fig. 27 Scatter plot and Spearman correlation between the main measure of overall experienced segregation and the alternative measure incorporating dwell time. Each point represents a neighbourhood (n=207,890 neighbourhoods), with the x-axis showing experienced segregation from the main analysis and the y-axis showing the adjusted measure accounting for median dwell time at activity sites. The texts show results of spearman correlation, and statistical significance was assessed using two-sided tests. The high correlation indicates the robustness of the main measure.

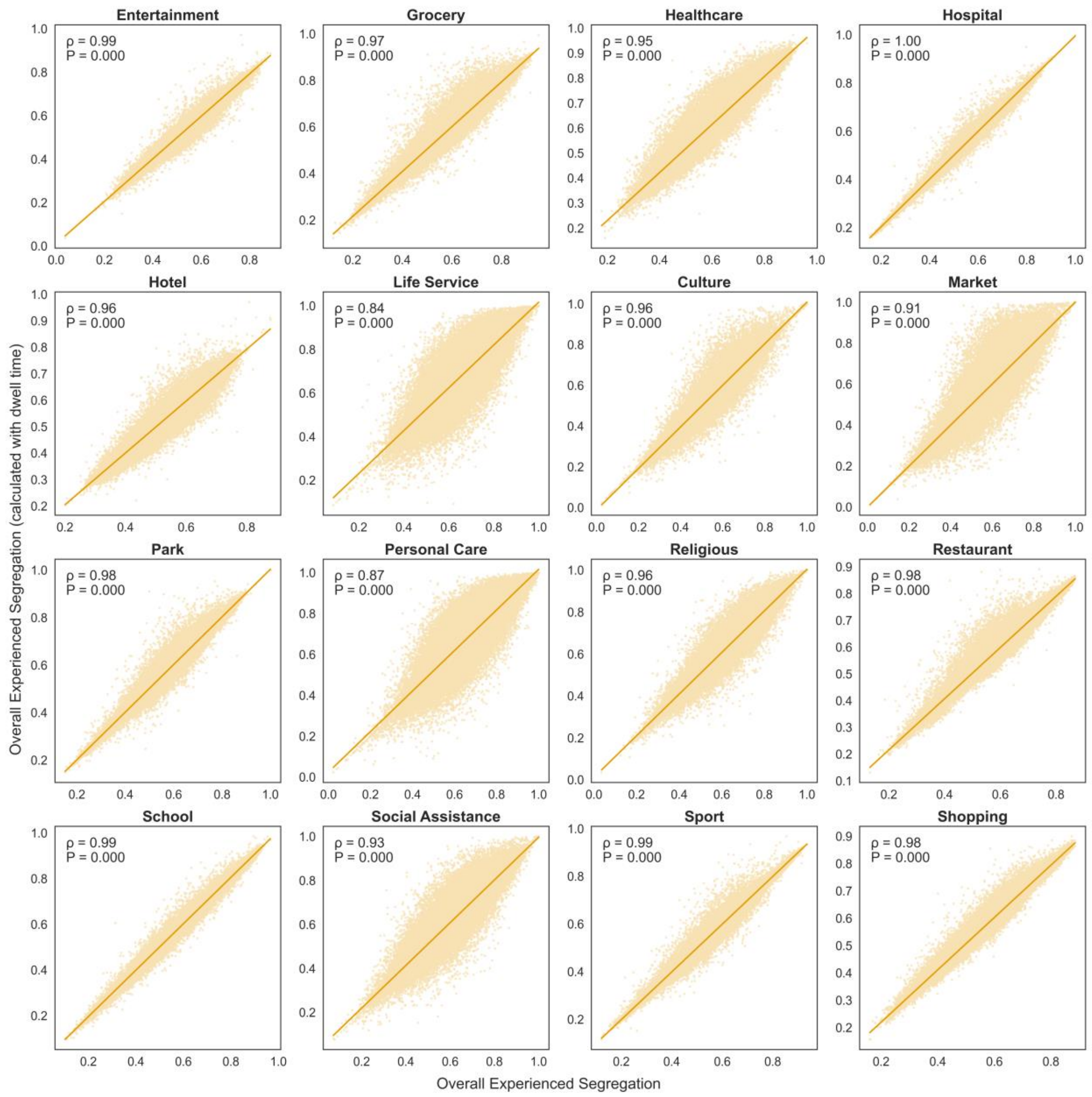

Supplementary Fig. 28 Scatter plot and Spearman correlation between the main measure of overall experienced segregation and the alternative measure incorporating dwell time by activity sites. Each point represents a neighbourhood: entertainment (n = 207,772 neighbourhoods), grocery (n = 207,884 neighbourhoods), healthcare (n = 207,882 neighbourhoods), hospitals (n = 207,879 neighbourhoods), hotels (n = 207,879 neighbourhoods), life service (n = 207,784 neighbourhoods), culture (n = 206,756 neighbourhoods), market (n = 206,159 neighbourhoods), park (n = 207,871 neighbourhoods), personal care (n = 207,432 neighbourhoods), religious (n = 207,836 neighbourhoods), restaurant (n = 207,889 neighbourhoods), school (n = 207,883 neighbourhoods), social assistance (n = 207,714 neighbourhoods), sport (n = 207,866 neighbourhoods), and shopping (n = 207,888 neighbourhoods). Noted that some neighbourhoods exhibit no mobility records for several types of activity sites. The x-axis showing experienced segregation from the main analysis and the y-axis showing the adjusted measure accounting for median dwell time at activity sites. The texts show results of spearman correlation, and statistical significance was assessed using two-sided tests. The high correlation indicates the robustness of the main measure.

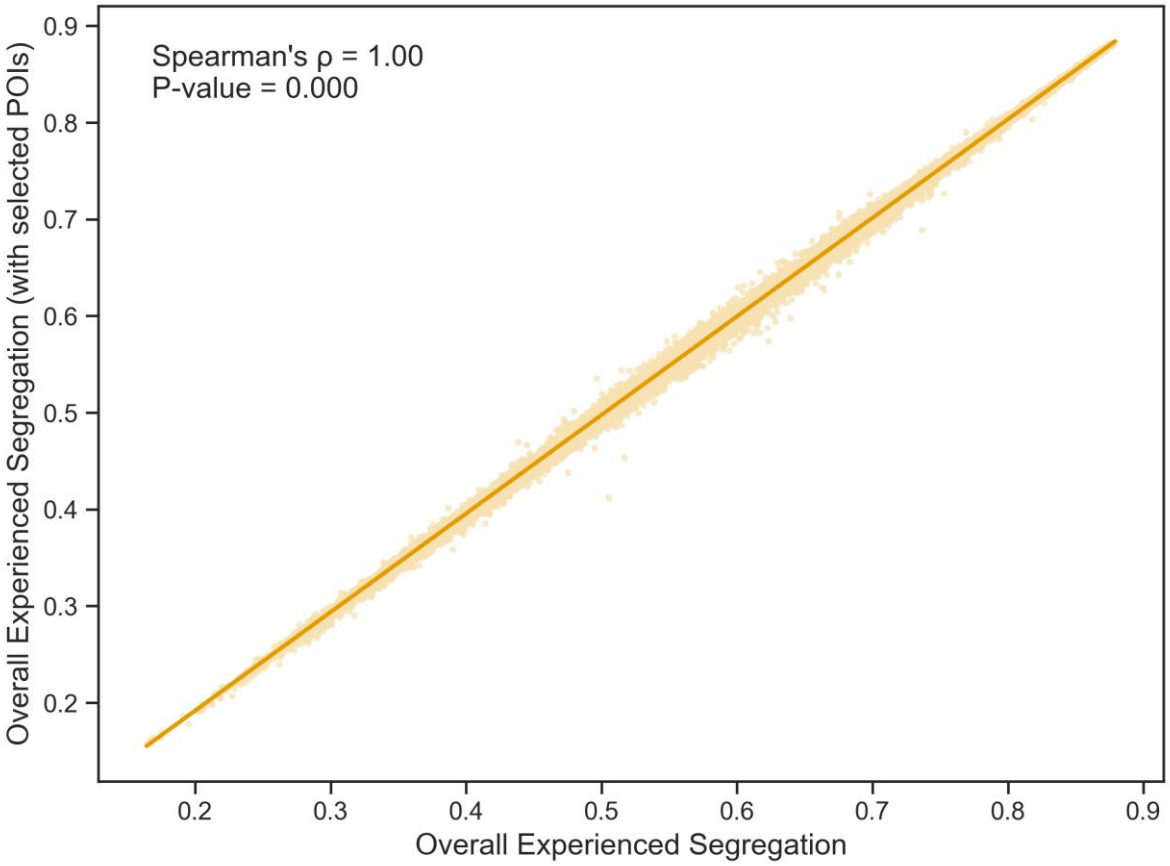

Supplementary Fig. 29 Scatter plot and Spearman correlation between the main measure of overall experienced segregation and the alternative measure based on selected types of activity sites. Each point represents a neighbourhood (n=207,890 neighbourhoods), with the x-axis showing experienced segregation from the main analysis and the y-axis showing the adjusted measure that includes only activity sites assumed to involve higher levels of social interaction. The texts show results of spearman correlation, and statistical significance was assessed using two-sided tests. The high correlation indicates the robustness of the main measure.

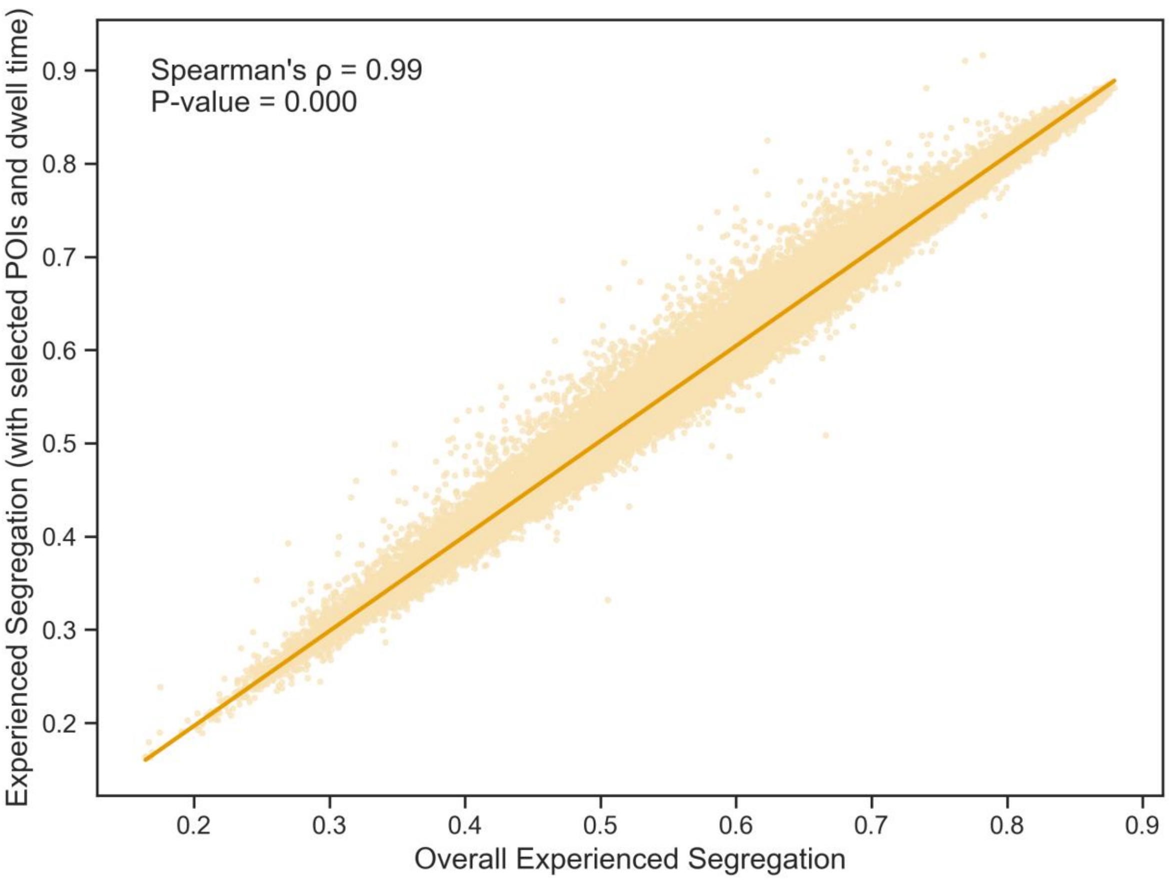

Supplementary Fig. 30 Scatter plot and Spearman correlation between the main measure of overall experienced segregation and the alternative measure that incorporates both selected types of activity sites and median dwell time. Each point represents a neighbourhood (n=207,890 neighbourhoods), with the x-axis showing experienced segregation from the main analysis and the y-axis showing the adjusted measure that includes only activity sites assumed to involve higher levels of social interaction and accounts for median dwell time at activity sites. The texts show results of spearman correlation, and statistical significance was assessed using two-sided tests. The high correlation indicates the robustness of the main measure.
