# Supplementary material for: Varying relationships between experienced income segregation and travel behaviour across neighbourhood social and urban contexts
Source: Nat Commun. 2025 Dec 18;16:11236. doi: 10.1038/s41467-025-66585-z (PMC12714732; doi:10.1038/s41467-025-66585-z)
Supplement: Supplementary file 2 — Reporting Summary [file 41467_2025_66585_MOESM2_ESM.pdf]

nature portfolio

Corresponding author(s): Yi Lu

Last updated by author(s): Oct 3, 2025

Reporting Summary

Nature Portfolio wishes to improve the reproducibility of the work that we publish. This form provides structure for consistency and transparency in reporting. For further information on Nature Portfolio policies, see our Editorial Policies and the Editorial Policy Checklist.

Statistics

For all statistical analyses, confirm that the following items are present in the figure legend, table legend, main text, or Methods section.

n/a	Confirmed	
☐	☒	The exact sample size (*n*) for each experimental group/condition, given as a discrete number and unit of measurement
☐	☒	A statement on whether measurements were taken from distinct samples or whether the same sample was measured repeatedly
☐	☒	The statistical test(s) used AND whether they are one- or two-sided<br>*Only common tests should be described solely by name; describe more complex techniques in the Methods section.*
☐	☒	A description of all covariates tested
☒	☐	A description of any assumptions or corrections, such as tests of normality and adjustment for multiple comparisons
☐	☒	A full description of the statistical parameters including central tendency (e.g. means) or other basic estimates (e.g. regression coefficient) AND variation (e.g. standard deviation) or associated estimates of uncertainty (e.g. confidence intervals)
☐	☒	For null hypothesis testing, the test statistic (e.g. *F*, *t*, *r*) with confidence intervals, effect sizes, degrees of freedom and *P* value noted<br>*Give P values as exact values whenever suitable.*
☒	☐	For Bayesian analysis, information on the choice of priors and Markov chain Monte Carlo settings
☒	☐	For hierarchical and complex designs, identification of the appropriate level for tests and full reporting of outcomes
☐	☒	Estimates of effect sizes (e.g. Cohen's *d*, Pearson's *r*), indicating how they were calculated

Our web collection on statistics for biologists contains articles on many of the points above.

Software and code

Policy information about availability of computer code

Data collection	No special software was used to collect the data.
Data analysis	Data preprocessing was performed by Python (version 3.12.7). Statistical analysis was conducted in R (version 4.1.1). Mapping was conducted in ArcGIS Pro (version 3.1.3). Main packages used in Python are: pandas (version 2.2.2), numpy (version: 1.26.4), scipy (version: 1.14.1), matplotlib (version: 3.9.2), seaborn (version: 0.13.2). Pakages used in R are: dplyer (version 1.1.1), tidyr (version 1.3.1), car (version 3.1.2), broom (version 0.7.9). All codes used to produce main and supplementary results in this study are available in the following repository: https://figshare.com/projects/Travel_Behaviour_and_Income_Segregation/266242.

For manuscripts utilizing custom algorithms or software that are central to the research but not yet described in published literature, software must be made available to editors and reviewers. We strongly encourage code deposition in a community repository (e.g. GitHub). See the Nature Portfolio guidelines for submitting code & software for further information.

Data

Policy information about availability of data

All manuscripts must include a data availability statement. This statement should provide the following information, where applicable:

- Accession codes, unique identifiers, or web links for publicly available datasets
- A description of any restrictions on data availability
- For clinical datasets or third party data, please ensure that the statement adheres to our policy

The mobility data was obtained from SafeGraph, which is commercially available and can be requested for research use (https://www.safegraph.com/pricing). Other data used in this study are open access. The NAICS codes for POIs classification were sourced from the official website for census bureau of the United States (https://www.census.gov/naics/). The socioeconomic data was derived from the American Community Survey (ACS) 5-Year Estimates for 2015 to 2019 (https://data.census.gov/). The RUCA codes for defining urbanicity levels were sourced from the USDA (https://www.ers.usda.gov/data-products/rural-urban-commuting-area-codes/). Shapefiles for mapping were obtained from the Topologically Integrated Geographic Encoding and Referencing System (TIGER) geodatabases (https://www.census.gov/geographies/mapping-files/time-series/geo/tiger-geodatabase-file.html). Data of access to public transit was derived from Smart Location Database provided by Environmental Protection Agency (EPA) of the United States (https://www.epa.gov/smartgrowth/smart-location-mapping). Source Data are provided in the following repository: https://figshare.com/projects/Travel_Behaviour_and_Income_Segregation/266242.

Research involving human participants, their data, or biological material

Policy information about studies with human participants or human data. See also policy information about sex, gender (identity/presentation), and sexual orientation and race, ethnicity and racism.

Reporting on sex and gender	N.A.
Reporting on race, ethnicity, or other socially relevant groupings	N.A.
Population characteristics	N.A.
Recruitment	N.A.
Ethics oversight	N.A.

Note that full information on the approval of the study protocol must also be provided in the manuscript.

Field-specific reporting

Please select the one below that is the best fit for your research. If you are not sure, read the appropriate sections before making your selection.

☐ Life sciences ☒ Behavioural & social sciences ☐ Ecological, evolutionary & environmental sciences

For a reference copy of the document with all sections, see nature.com/documents/nr-reporting-summary-flat.pdf

Behavioural & social sciences study design

All studies must disclose on these points even when the disclosure is negative.

Study description	Using a dataset of 1.2 billion mobility records from the contiguous United States, we quantify experienced income segregation and examined its relationships with travel distance and diversity across neighbourhoods with different social and urban contexts. We find that longer travel distances and more diverse destinations are associated with less experienced segregation for least affluent neighbourhoods, especially in less urbanized areas. Our findings underscore the need for urban planning and transport interventions to increase mobility and social integration opportunities for residents from socially disadvantaged neighbourhoods and address the potential adverse social consequences of localized living models.
Research sample	The analysis utilizes mobility dataset provided by SafeGraph, which includes all available weekly data throughout 2019 across the contiguous United States. To quantify income segregation, our study included 1,226,450,275 Origin-Destination (OD) pairs from 208,356 census block groups (CBGs) to 3,227,842 points of interest (POIs). After excluding cases with missing information (e.g., urbanicity classification), a total of 207,890 CBGs were retained for the subsequent analyses, including 167,780 CBGs classified as metropolitan areas, 20,580 CBGs as micropolitan areas, 10,847 CBGs as small towns, and 8,683 CBGs as rural areas.
Sampling strategy	We did not perform sampling, sample size was determined by the size of SafeGraph dataset.
Data collection	Mobility data was collected by SafeGraph. The socioeconomic data was derived from the American Community Survey (ACS) 5-Year Estimates for 2015 to 2019 (https://data.census.gov/). The RUCA codes for defining urbanicity levels were sourced from the USDA (https://www.ers.usda.gov/data-products/rural-urban-commuting-area-codes/). Shapefiles for mapping were obtained from the Topologically Integrated Geographic Encoding and Referencing System (TIGER) geodatabases (https://www.census.gov/geographies/

	mapping-files/time-series/geo/tiger-geodatabase-file.html). Data of access to public transit was derived from Smart Location Database provided by Environmental Protection Agency (EPA) of the United States (https://www.epa.gov/smartgrowth/smart-location-mapping).
Timing	Mobility data were obtained for the year 2019. Socioeconomic data were derived from the 2015–2019 ACS 5-Year Estimates, reflecting conditions as of 2019. To ensure consistency in spatial boundaries, urbanicity levels were defined using the 2010 Rural–Urban Commuting Area (RUCA) codes, as geographic boundaries were updated in the 2020 census. Data on access to public transit were sourced from the Smart Location Database, using the version released in 2021, which remains the latest available for download.
Data exclusions	No data were excluded.
Non-participation	No participation was involved in this study.
Randomization	This study did not involve experimental conditions. Thus, this is not applicable.

Reporting for specific materials, systems and methods

We require information from authors about some types of materials, experimental systems and methods used in many studies. Here, indicate whether each material, system or method listed is relevant to your study. If you are not sure if a list item applies to your research, read the appropriate section before selecting a response.

Materials & experimental systems

n/a	Involved in the study
☒	☐ Antibodies
☒	☐ Eukaryotic cell lines
☒	☐ Palaeontology and archaeology
☒	☐ Animals and other organisms
☒	☐ Clinical data
☒	☐ Dual use research of concern
☒	☐ Plants

Methods

n/a	Involved in the study
☒	☐ ChIP-seq
☒	☐ Flow cytometry
☒	☐ MRI-based neuroimaging

Plants

Seed stocks	N.A.
Novel plant genotypes	N.A.
Authentication	N.A.
